# A new framework for characterization of poroelastic materials using indentation

**DOI:** 10.1016/j.actbio.2019.11.010

**Published:** 2020-01-15

**Authors:** Mohammad Hadi Esteki, Ali Akbar Alemrajabi, Chloe M. Hall, Graham K. Sheridan, Mojtaba Azadi, Emad Moeendarbary

**Affiliations:** a Department of Mechanical Engineering, Isfahan University of Technology, Isfahan 84156-83111, Iran; b Department of Mechanical Engineering, University College London, London, United Kingdom; c School of Pharmacy and Biomolecular Sciences, University of Brighton, Brighton, United Kingdom; d School of Life Sciences, Queen's Medical Centre, University of Nottingham, Nottingham, United Kingdom; e School of Engineering, College of Science and Engineering, San Francisco State University, San Francisco, CA 94132, United States; f Department of Biological Engineering, Massachusetts Institute of Technology, Cambridge, MA, United States

**Keywords:** Poroelasticity, Indentation, Relaxation time, Finite element model, Hydrogels, Atomic force microscopy

## Abstract

To characterize a poroelastic material, typically an indenter is pressed onto the surface of the material with a ramp of a finite approach velocity followed by a hold where the indenter displacement is kept constant. This leads to deformation of the porous matrix, pressurization of the interstitial fluid and relaxation due to redistribution of fluid through the pores. In most studies the poroelastic properties, including elastic modulus, Poisson ratio and poroelastic diffusion coefficient, are extracted by assuming an instantaneous step indentation. However, exerting step like indentation is not experimentally possible and usually a ramp indentation with a finite approach velocity is applied. Moreover, the poroelastic relaxation time highly depends on the approach velocity in addition to the poroelastic diffusion coefficient and the contact area. Here, we extensively studied the effect of indentation velocity using finite element simulations which has enabled the formulation of a new framework based on a master curve that incorporates the finite rise time. To verify our novel framework, the poroelastic properties of two types of hydrogels were extracted experimentally using indentation tests at both macro and micro scales. Our new framework that is based on consideration of finite approach velocity is experimentally easy to implement and provides a more accurate estimation of poroelastic properties.

**Statement of significance:**

Hydrogels, tissues and living cells are constituted of a sponge-like porous elastic matrix bathed in an interstitial fluid. It has been shown that these materials behave according to the theory of ‘poroelasticity’ when mechanically stimulated in a way similar to that experienced in organs within the body. In this theory, the rate at which the fluid-filled sponge can be deformed is limited by how fast interstitial fluid can redistribute within the sponge in response to deformation. Here, we simulated indentation experiments at different rates and formulated a new framework that inherently captures the effects of stimulation speed on the mechanical response of poroelastic materials. We validated our framework by conducting experiments at different length-scales on agarose and polyacrylamide hydrogels.

## Graphical abstract

**Figure:**
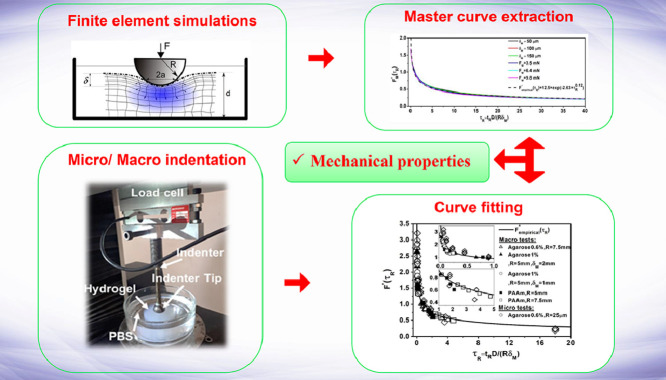

## 1 Introduction

Due to their practical applications in biomedicine and bioengineering, substantial efforts have been made to characterize the unique biophysical properties of soft and hydrated materials such as polymers, colloids, amphiphilics, membranes, micelles, emulsions, dendrimers, liquid crystals and polyelectrolytes [1]. The mechanical responses of a wide range of hydrated materials has been found to be both time- and length- scale dependent and this is best described by poroelastic theory [2], [3], [4], [5], [6], [7], [8], [9], [10], [11], [12]. The one-dimensional theory of the consolidation of a water-saturated geo-material was first developed by Terzaghi and subsequently extended by Biot to introduce the 3D deformation of the elastic porous medium bathed in fluid [13], [14], [15]. Despite significant progress in the development of poroelastic theory and advances in computational modeling, the field still lacks an inclusive framework to estimate the poroelastic properties of soft hydrated materials from experimental measurements. Extracting accurate mechanical behavior of soft materials will accelerate biomedical research in fields such as cell and tissue regeneration, drug delivery, hygiene products, and microfluidic technology [1,16].

**Fig. 1:**
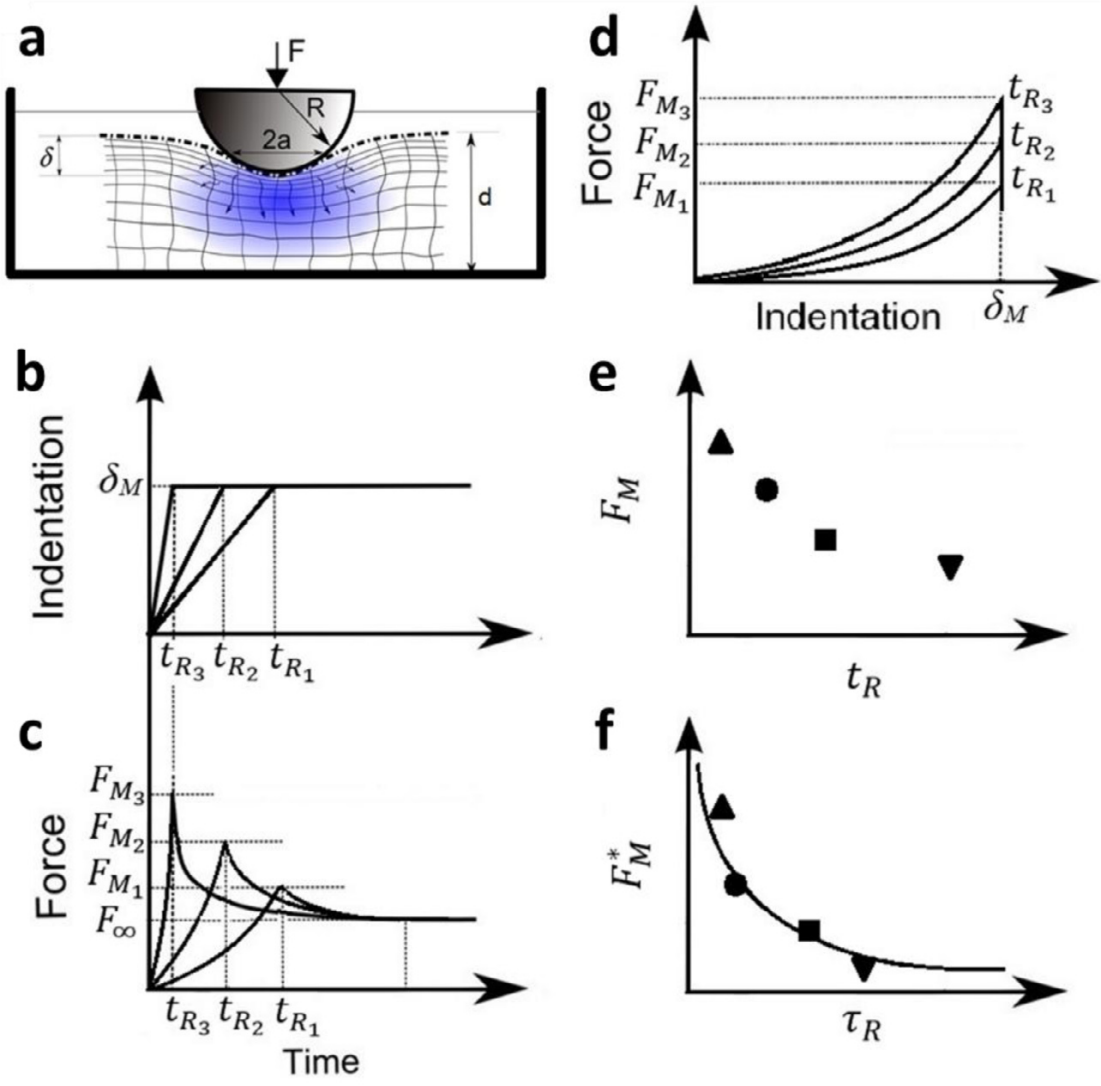
(a) Schematic of spherical indentation test and the relevant geometrical parameters: indentation depth *δ*, indenter radius *R*, material thickness *d* and contact radius *a*. (b, c, d) Force–relaxation tests consist of ramp and hold phases separated through rise time *t_R_*. (b) The indentation depth versus time for different rise times *t_R_*: in the ramp phase the indentation depth increases linearly with time until reaching a maximum depth of *δ_M_* at time *t_R_* that is kept constant during hold phase. (c) The force versus time for different rise times *t_R_*: the indentation depth is kept constant during the hold phase and the maximum force *F_M_* that is achieved during ramp phase relaxes to a fully relaxed force *F_∞_* after prolonged time. Considering different rise times and keeping the maximum indentation depth constant yield different approach velocities and result in different maximum forces. (d) Plots of the force against indentation depth for different *t_R_*. (e) Maximum forces *F_M_* emerged as a result of different approach velocities were plotted against the rise times *t_R_*. (f) Normalized maximum force versus normalized rise time: appropriate normalization of maximum forces and rise times lead to a master curve as proposed in this study.

Indentation tests at micro and macro scales conducted alongside computational modeling have been the most common framework for characterizing the mechanical behavior of soft hydrated materials [8,^9^,[17], [18], [19]. Recently, the elastic and transport properties, such as shear modulus *G* and permeability *κ* of biological tissues, cells and various hydrogels [17], [18], [19], [20], [21], [22], [23], [24], [25], [26], [27], [28] have been estimated, using the poroelasticity framework, by analyzing the ramp and hold phases of indentation experiments (Fig. 1). In most studies, instantaneous indentation has typically been assumed and elasticity was estimated by fitting the force–indentation curve (Fig. 1(d)) while poroelastic diffusion constant was estimated by analyzing the relaxation curve (Fig. 1(c)). Moreover, how the length scale affects relaxation curves has been studied by altering parameters such as the indentation depth, indenter geometry, poroelastic material structure and thickness [17,^18^,^22^,^23^,[29], [30], [31]. To estimate the poroelastic parameters the relaxation master-curves were derived by normalizing the relaxation force *F(t)* (via [*F(t)-F_∞_*]/[*F_M_- F_∞_*]) and time *t* (via *t*/*a^2^*) considering stepwise indentation, i.e. the maximum force *F_M_* achieved at infinitely fast indentation velocity (*V_app_* ∼ ∞ or *t_R_* ∼ 0, Fig. 1) [17,^18^,^21^,^22^,^25^,32]. However, experimentally it is not possible to achieve such an ideal condition (*t_R_* >> 0) due to the limited ability of the instruments to provide accurate step load. Moreover, using fast approach velocities may lead to instability and unwanted oscillations. Therefore, in principal, extracting poroelastic parameters using the master curves that are derived under the instantaneous loading (*t_R_* ∼ 0) condition may lead to significantly unrealistic values, particularly when the experimentally set rise time (*t_R_*) is not drastically smaller than the poroelastic relaxation time (*τ_p_*). A few studies have examined effects of ramp velocity on the hold phase [33] and mostly in creep indentation experiments on poroelastic materials [34,35]. However, they lack building a master–curve framework that considers effects of rise time.

To overcome these drawbacks, here we ran a series of finite element (FE) simulations and extended the analysis of poroelastic indentation framework by investigating the effects of rise time *t_R_* on ramp and hold phases. We introduce a novel approach to normalize rise time and the associated maximum force *F_M_* (Fig. 1(e)) and extract a dimensionless master curve (Fig. 1(f)). Our approach allows estimation of poroelastic parameters of soft hydrated materials using indentation without the need for computational modeling or numerical simulations. To validate and evaluate the capabilities of our novel framework, we conducted indentation tests on two hydrogels with dominant poroelastic behavior (i.e. agarose and polyacrylamide hydrogels) at micro and macro scales and extracted their poroelastic properties using our novel method. Agarose hydrogels are widely used in cell and tissue culture systems, cartilage repair devices, and magnetic resonance elastography [36], [37], [38]; whilst polyacrylamide (PAAm) hydrogels have a wide range of biomedical and bioengineering applications, including electrophoresis, enzyme immobilization, drug delivery, smart biomaterials, reconstructive surgery and extracorporeal toxin removal modalities [39].

## 2 Materials and methods

### 2.1 The FEM method

The two-dimensional axisymmetric Finite Element model (FEM) of indentation of poroelastic hydrogel specimens was constructed in ABAQUS (version 2018). The geometry and boundary conditions of the discretized model are shown in Supplementary materials (Fig. S1). To minimize the effects of edges on force relaxation, the hydrogel was modeled as a cylindrical disk with 30 mm radius and 60 mm thickness and indented with an infinitely rigid indenter of size *R* = 10 mm. The contact between the indenter and the hydrogel was considered to be frictionless and impermeable and no slip condition was set for the bottom surface. The pore pressure was set to zero at all surfaces of the specimen (excluding the indenter contact surface) to simulate free draining of the interstitial fluid from the porous matrix (Fig. S1). The FE simulation consists of both ramp and hold steps. First, the ramp step involves indentation of the material surface with a predefined rise time and specified displacement in the *z*-direction using a spherical indenter. Next, the hold step is the phase in which the force reaction is relaxed. As the first approximation, we considered a linear isotropic poroelastic constitutive model in the FE simulations [17,^18^,^22^,23] while nonlinear geometry and structured mesh options were employed to capture the possible effects of large deformations within the vicinity of the contact area. While investigating the effects of large deformations and material nonlinearity is beyond the scope of our work, recent research [40], [41], [42] suggests that up to *δ/R* ∼ 0.6 of the Hertz load displacement relationship still holds true. Therefore, our linear assumption provides a good estimation of poroelastic parameters as a first approximation. Furthermore, we ran several simulations considering the neo-Hookean model of poroelasticity for comparison with the linear framework.

The poroelastic domain was discretized with the quadratic quadrilateral CAX8P standard element and the mesh sensitivity was assessed by refining the structured mesh size automatically until the results were independent of element size. To account for the localized deformation of the contact surface, it is essential that the density of nodes close to the contact region is high enough. The implicit finite element solver, ABAQUS/Standard commercial finite element code, was applied to work out the indentation test problem. The implicit FEM predictions in Abaqus employ automatic (small) time step with Newton-type iterations. In this study, the SOILS analysis in Abaqus was used to model the response of the poroelastic samples in the indentation tests. In the ramp phase (0 < *t* < *t_R_*), set thresholds of either maximum indentation depth (*δ_M_*) or maximum set force (*F_M_*) were considered and the resultant indentation *δ*(*t*) and the relaxation force *F(t)* functions were calculated. The sensitivity of the FE simulations to domain size and mesh element numbers were checked. A mesh convergence study was then performed in the ramp phase by decreasing the mesh size until the error of the estimated maximum force *F_M_* at the indenter tip for fine mesh was less than 0.1% of the coarse mesh. In addition, the optimum sizes of the domain in lateral and vertical directions were calculated by increasing the size in both directions until the error in the maximum force, *F_M_*, was less than 0.1%. For SOILS analysis, a tolerance for maximum pore pressure change per increment was required. The pore pressure and fully relaxed force *F∞* are the main effective parameters of the poroelastic simulations. The convergence of the computational program for each case (defined mesh and geometry size) was achieved by decreasing the pore pressure rate change until the error in the fully relaxed force *F∞* is minimized (Fig. S1). The FE simulation parameters (indenter size and indentation depth) and poroelastic properties (shear modulus, diffusion coefficient and Poisson ratio) were chosen from the previously experimentally-derived properties for indentation of a PAAm hydrogel [22], as tabulated in Table S1. The choice of hyperelastic parameters, as tabulated in Table S2, were from [22,43].

### 2.2 Hydrogel preparation

Because agarose and PAAm hydrogels display prototypical features of a predominantly poroelastic material, both gel types were used for indentation testing. The 0.6% and 1% agarose hydrogels were prepared separately by either suspending 0.6 g or 1 g agarose powder (Invitrogen, Carsbad, CA) in 100 mL phosphate buffered saline (PBS) followed by heating to 90 °C until the solution became clear. The solution was cast into 33 mm circular culture dishes and left at room temperature for 2 h to cure. The gel was maintained fully hydrated in PBS and cooled to 4 ^o^C for 48 h to obtain uniform swelling and an equilibrium state before testing. To avoid unexpected changes in mechanical properties of gels, the temperature and cooling time were carefully controlled [44].

The 6% PAAm gels were prepared by dissolving 30% (w/v) acrylamide (Bio-Rad, UK) stock solution with a 29:1 acrylamide to bis-acrylamide ratio in 100 mL distilled water. After degassing for 20 min, the gels were synthesized by adding 10 % w/v ammonium persulfate solution (APS) and tetramethylethylenediamine (TEMED) as the initiators [45]. The stock solution was cast into 30 mm radius Petri dishes and left at room temperature in anaerobic conditions for 30 min. After solidification, PAAm gels were carefully separated from the mold and were kept hydrated with PBS at 23 ^o^C for a further two weeks to achieve an equilibrium-swelled state prior to testing.

### 2.3 Atomic force microscopy indentation

The microscale indentation experiments were run by applying the JPK Atomic Force Microscope (AFM) Nanowizard-CellHesion (JPK instruments, Berlin, Germany) interfaced to an inverted optical fluorescence microscope. The AFM cantilevers (RFESP-75, Bruker, Karlsruhe, Germany) were prepared by gluing spherical beads to the cantilever tip with UV curing glue (UV curing, Loctite, UK). Cantilever spring constants were determined prior to gluing the beads by applying the thermal noise method implemented in the AFM setting (JPK SPM, JPK instruments). Prior to any indentation test, the sensitivity of the cantilever was adjusted by measuring the slope of force–distance curves acquired on glass. The nominal spring constant of 2.5–3 N m^−1^ and 25 µm radius glass beads (Cospheric) were used to indent the softest hydrogels (0.6% agarose) of 30 mm diameter and 5 mm thickness, submerged in PBS solution.

The AFM cantilever was pressed into the hydrogel surface considering eight defined approach velocities (base of the AFM cantilever velocities: *V* = 1, 10, 20, 40, 80, 160, 320 and 640 µm · s^−1^) until reaching three target forces (*F_M_* = 500, 1000 and 1600 nN). This yielded 18 different maximum indentation depths, *δ_M_*, which were estimated by finding the contact point [46]. From the point that the indenter contacts the surface of the sample, the displacement of the cantilever base is translated into the indentation depth and the deflection of cantilever. In our experiments, the choice of cantilever stiffness was such that the deflection of the cantilever is always less than 10% of the indentation depth. Consequently, the approach velocity of the spherical indenter can be assumed to be approximately the same as the speed of the cantilever base.

### 2.4 Macroscale indentation

The macroscale indentation experiments were performed by applying a uniaxial tensile tester machine with a cross-head position resolution of 1 µm equipped with ±10 N load cell with a force resolution of 1 mN. Indentation tests were run by applying a rigid stainless steel spherical indenter radius of either *R* = 5, 7.5 or 10 mm. To minimize substrate and geometrical effects, we ensured that the hydrogel thickness and size for macroindentation tests were at least ten times larger than the contact area and indentation depth, respectively [47]. The 6% PAAm and the 0.6% and 1% agarose hydrogels, with a minimum thickness of 30 mm and radius of 60 mm were fully submerged in PBS during indentation experiments. To avoid slip between the hydrogel and the substrate, hydrogel samples were attached to the bottom of the test chamber by cyanoacrylate prior to submerging in PBS. Two samples were selected for macroscale indentation tests. On each hydrogel, four measurements were taken and each were spaced at least 2 cm apart from each other and the sample edge to minimize any overlap [24].

The indenter was lowered onto the hydrogel surface to reach a small preset force while the displacement on the tester machine was adjusted. When the indenter contacted the hydrogel surface, a positive slope of the force–displacement response was recorded and while the indentation depth *δ* increased, the indenter was pressed further into the hydrogel to reach a defined maximum depth *δ_M_* leading to a maximum reaction force *F_M_* recorded via the tester. For each predefined δ*_M_* and rise time *t_R_*, leading to a specific *F_M_*, the relaxation of the force versus time was recorded.

For each hydrogel type, the indenter size and the indentation depth were chosen to maximize the sensitivity of tester machine. A spherical indenter of size *R* = 7.5 mm was used to conduct force–relaxation experiments on the 0.6% agarose hydrogels (*δ_M_*=3 mm and *t_R_* = 3, 30, 120 and 300 s), the PAAm samples (*δ_M_* = 1, 2 and 3 mm and *t_R_* = 1000, 2000 and 3000 s) and size R = 5 mm for 1% agarose hydrogels (*δ_M_* = 2 mm and *t_R_*=50, 150, 200 and 400 s). Indenters of size *R* = 5 and 10 mm were also used for 1% agarose hydrogels (*δ_M_* = 0.5, 1 and 1.5 mm and *t_R_*=100, 200 and 300 s). The relaxation tests were complete when the force reached a steady-state flat value.

## 3 Results and discussion

### 3.1 FEM analysis: effects of approach velocity and contact size

**Fig. 2:**
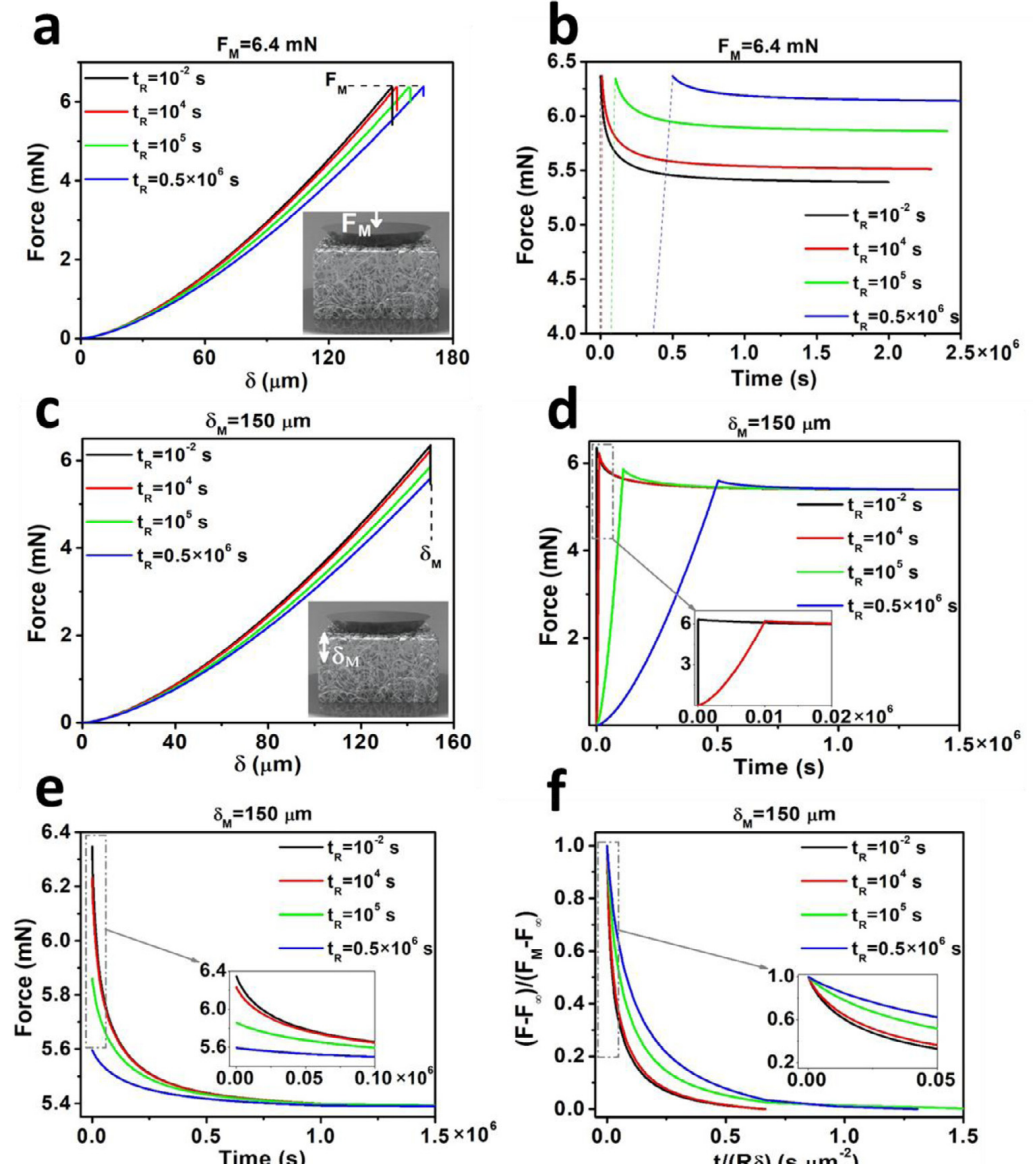
Force–indentation (a, c) and force–relaxation (b, d) curves generated from FEM considering four approach velocities = 1.5 × 10^4^, 1.5 × 10^−2^, 1.5 × 10^−3^ and 3 × 10^−4^$\;\mu m\cdot s^{-1}$. In (a, b), a constant set force of *F_M_* = 6.4 mN was used while in (c––f), a maximum indentation depth of *δ_M_* = 150 µm was considered. (e) Only force–relaxation part of curves in (b) were considered with the initial time of relaxation curves set to zero. (f) Normalization of the force–relaxation curves with the methodology introduced in [17,^18^,22] did not lead to the collapse of relaxation curves into a single curve, since normalization does not take into account the effect of approach velocity.

Poroelastic indentation tests were simulated by FEM and different approach velocities were considered (i.e. different rise times *t_R_*). Similar to micro and macro scale experiments, the stress-relaxation tests can be achieved by either considering a preset maximum indentation depth (*δ_M_*, as in the case of macro-indentations) or a preset maximum force (*F_M_*, as in the case of AFM micro-indentation tests). Based on the material properties of Table S1, the macro-indentations were simulated considering either a maximum set force of *F_M_* = 6.4 mN (Fig. 2(a)) or a maximum indentation depth of *δ_M_* = 150 µm (Fig. 2(c)). Force indentation (*F–δ*) and force–relaxation (*F–t*) are plotted in Fig. 2. Force–relaxation curves reach a final relaxation force of *F_∞_* ∼ 5.5 mN after *t_∞_* ∼ 1.4 × 10^6^ s. The results indicate that the shape of both *F–δ* and *F–t* curves are strongly dependent on rise time *t_R_*, i.e. at high indentation approach velocities, the fluid in the gel structure remains at its equilibrium state during the ramp phase and it only redistributes shortly after reaching the maximum indentation depth. This leads to a sudden and sharp relaxation of the force whilst at slow approach velocities the fluid redistributes during the ramp phase, leading to lower levels of relaxation in the hold phase.

To test whether previously introduced normalization methods are capable of capturing the effects of approach velocity, only force–relaxation curves in Fig. 2(d) were considered and plotted against time. Here, the initial time was set to zero (Fig. 2(e)). Subsequently, curves in Fig. 2(e) were normalized using normalized force [*F(t)–F_∞_*]/[(*F_M_–F_∞_*] and normalized time [*t*/*Rδ*] as described in [17,^18^,22]. This method of normalization (Fig. 2(f)) did not result in the collapse of curves into a single master curve which indicates that the *t_R_* or approach velocity must be considered as an important criterion, as ignored in recent works [17,^18^,^21^,^22^,^25^,32], for analysis of the poroelastic materials and the extraction of poroelastic parameters.

### 3.2 Analysis of constant effective approach velocity

#### 3.2.1 FEM analysis

**Fig. 3:**
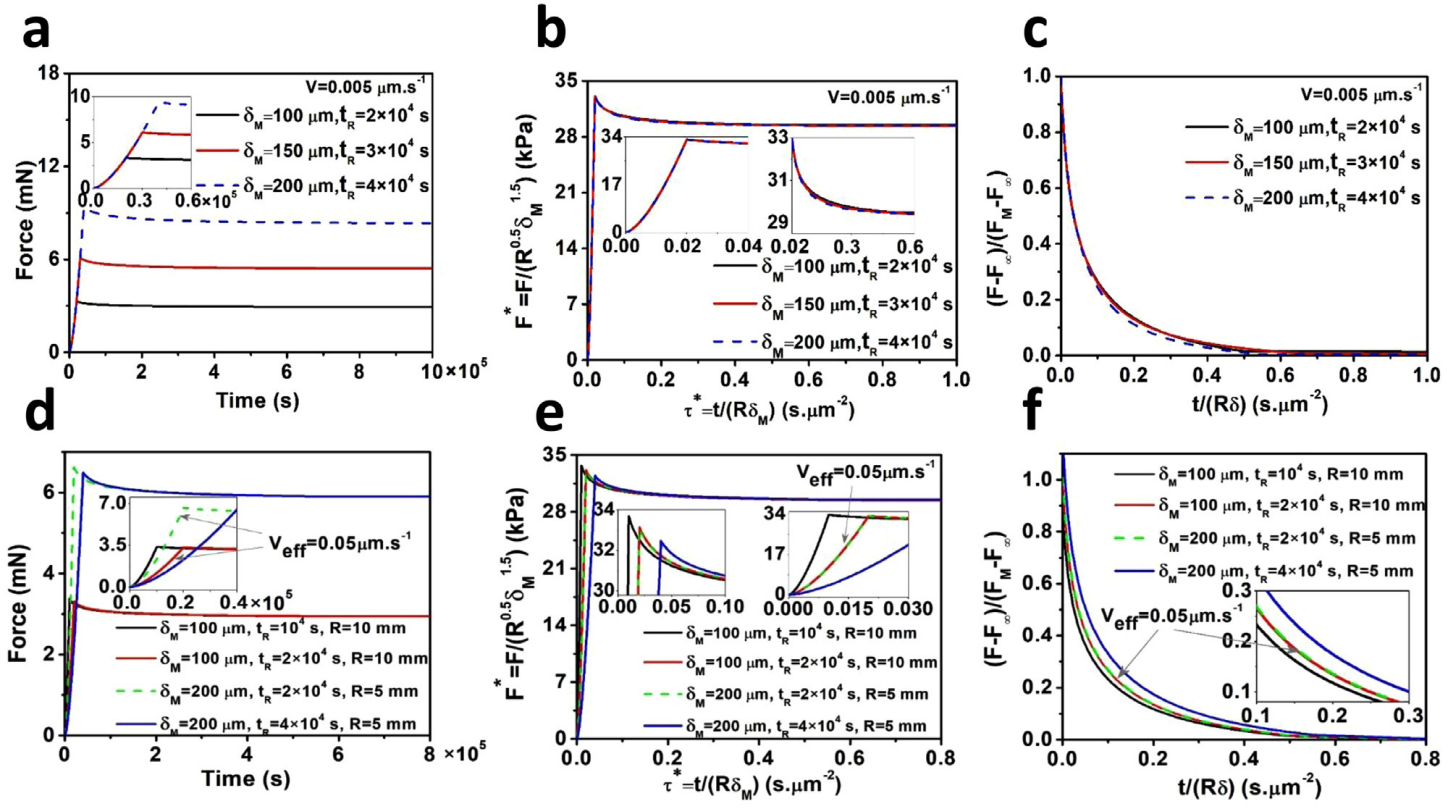
Non-normalized force–indentation and force–relaxation (a, d) and their normalization with two different methods shown in (b, e) and in (c, f). (a) The indentation depth *δ_M_* and rise time *t_R_* were varied proportionally to keep the approach velocity $V=\delta_{M}/t_{R}$ constant, leading to collapse of all curves into a single curve after implementing appropriate force and time normalizations as shown in (b, c). (d) Effects of varying indentation depth, rise time and indenter radius. (e, f) Only curves with the same $V_{eff}=\surd \left(R\delta_{M}\right)/t_{R}$ collapsed onto each other after normalization.

The conditions under which previously proposed and validated methods of force–relaxation normalization were next investigated [17,^18^,22]. First, we considered a constant approach velocity of $V=\delta_{M}/t_{R}=0.005\;\mu m\cdot s^{-1}$ and conducted FEM simulations of macro-indentation tests using the hydrogel parameters presented in Table S1. Three indentation depths were modeled: $\delta_{M}=100,\;150,\;200\;\mu m$. Considering a constant velocity yields rise times of *t_R_* = 2 × 10^4^, 3 × 10^4^, and 4 × 10^4^ s, respectively. The results of these simulations are shown in Fig. 3(a). Normalization of time with $\tau^{*}=t/R\delta_{M}$ and force with either $F^{*}\left(t\right)=F\left(t\right)/\;\left(R^{0.5}\delta_{M}^{1.5}\right)$ or $F^{*}\left(t\right)=\left[F\left(t\right)-F_{\infty }]/[F_{M}-F_{\infty }\right]\;$leads to the collapse of three curves into a single curve (Fig. 3(b) and (c)).

Next, a set of 4 FEM simulations were run for indenters of size *R* = 5 mm and 10 mm, indentation depths of $\delta_{M}=100\;\mu m\;\text{and}\;200\;\mu m,$ and approach velocities of $V=0.01\;\mu m.\;s^{-1}\text{and}\;0.005\;\mu m.\;s^{-1}$ as shown in Fig. 3(d). We defined the effective approach velocity as $V_{eff}=\surd \left(R\delta_{M}\right)/t_{R}$ and found that only normalized curves (described above in the methods) with the same *V_eff_* can be overlaid onto each other. Taken together, these results indicate that length scale needs to be considered in the approach phase, in addition to the relaxation phase, and previously extracted master curves are only valid under the assumption of a constant effective approach velocity.

#### 3.2.2 Experimental validation

The load–relaxation curves generated from the micro-indentation tests performed on 0.6% agarose hydrogels using a 25 µm indenter at three different approach velocities (i.e. *V* = 80, 10, 1 µm s^−1^) and three set target forces (i.e. 500, 1000, 1600 nN), are shown in Fig. S3a,b,c. The load–relaxation curves obtained from the macroscale indentation of PAAm hydrogels at *V* = 0.001 mm s^−1^ and *δ_M_* = 1, 2, 3 mm and from the 1% agarose hydrogels at *V* = 0.01, 0.005 mm s^−1^ and *δ_M_* = 0.5, 1, 1.5 mm, using indenters of different sizes (i.e. *R* = 5, 7.5, 10 mm) are shown in Figs. S4a, b and S5a, b, respectively. As observed in Figs. S3d, e, f, S4c,d and S5d,e, the relaxation curves collapse into single curves after normalization of the force with $F^{*}\left(\tau \right)=F\left(t\right)/\;\left(R^{0.5}\delta_{M}^{1.5}\right)\;$and the time with$\;\tau^{*}=t/R\delta_{M}$ and considering a constant approach velocity and indenter size. To evaluate the contribution of effective velocity, the experiments were run for different indenter sizes and approach velocities at the same *t_R_*, Fig. S5c. Despite the curves in Fig. S5c. being obtained using different approach velocities (velocities *V* = 0.01, 0.005 mm s^−1^), the normalized curves collapsed into a single curve since the effective approach velocity for these curves are the same, i.e. *V_eff_* = 0.022 mm s^−1^ (Fig. S5f). Taken together, our results show that the normalization of force–time curves leads to the collapse of curves into a single curve, but only under the condition of constant effective approach velocity$\;V_{eff}=\surd \left(R\delta_{M}\right)/t_{R}$.

### 3.3 Extraction of a novel master curve

**Fig. 4:**
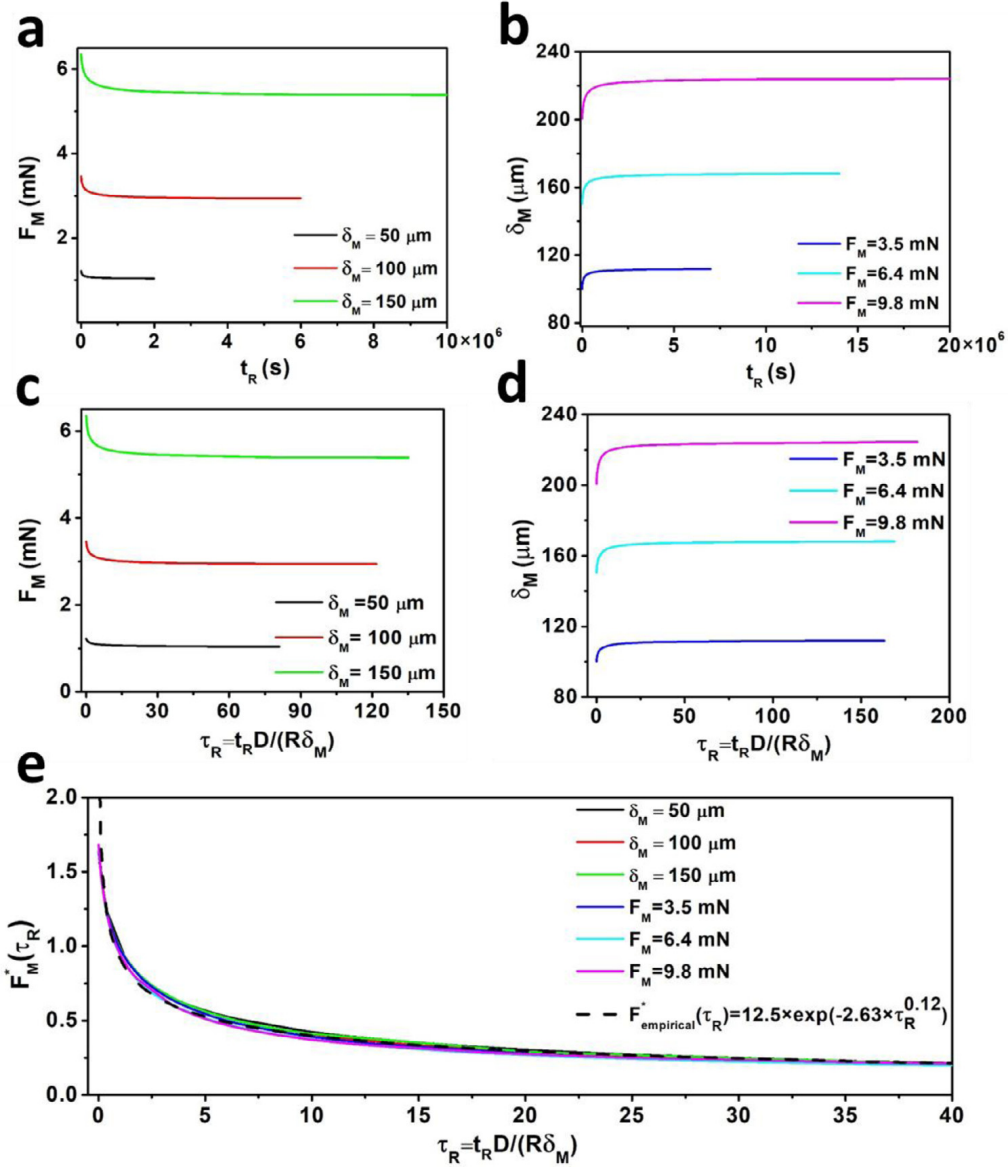
The novel normalization framework. (a) Plots of *F_M_* against *t_R_* for a set of three maximum indentation depths. (b) Plots of *δ_M_* against *t_R_* for a set of three maximum forces. (c, d) Normalization of timescale in curves (a) and (b) using $\tau_{R}=\frac{Dt_{R}}{R\delta_{M}}$ . (e) Normalization of both *F_M_* according to Eq. (1) and *t_R_* according to $\tau_{R}=\frac{Dt_{R}}{R\delta_{M}}$ led to the collapse of all curves into a single master curve. This master curve can be captured with a simple Eq. (4) and is valid for all poroelastic materials independent of indentation geometry.

Next, to construct a general framework, we performed a series of simulations (*N* = 2200) and investigated the effects of rise time on either the resultant maximum force or the maximum indentation depth. In other words, for each *t_R_*, a preset maximum indentation depth or a preset maximum force was considered, and the resultant maximum force or maximum indentation depth were estimated via FEM. The estimated F_M_, as a function of *t_R_* for the three preset maximum indentation depths (δ*_M_* = 50, 100 and 150 µm), are shown in Fig. 4(a)., whilst the estimated *δ_M_*, as a function of *t_R_* for three preset maximum forces (*F_M_* = 3.5, 6.4 and 9.8 mN), are shown in Fig. 4(b).

During deformation of a poroelastic material, the dominant mechanism for both pressurization and relaxation relies on the ability of the interstitial fluid to infiltrate through the pores of the solid phase. The hallmark of poroelastic mechanical behavior is that the length scale *L* for the pore pressure propagation and the timescale *t_p_* for the pore pressure relaxation are correlated through *t_p_* ∼ *L*^2^/*D*, where *D* is the poroelastic diffusion coefficient [17,22]. The presence of a characteristic length scale *L* is a unique feature of poroelastic mechanical responses. While the relaxation timescale of poroelastic material critically relies on the length scale, as has been extensively discussed particularly in the context of relaxation curves [48], the time scale of pressurization phase is also modulated by the length scale. Therefore, in the context of indentation tests, pressurization (and subsequently relaxation) would be weak (or does not occur at all) if the time scale of the ramp were to be significantly larger than *L*^2^/*D*. In other words, during indentation tests, we expect the relaxation phase to exhibit a more pronounced poroelastic signature when the rise time of the ramp phase is significantly shorter than the poroelastic relaxation timescale *t_R_* ≪ *t_p_* ∼ *L*^2^/*D*. The length scale for fluid redistribution/ pore pressure pressurization in spherical indentation tests is the contact radius $L∼a=\sqrt{R\delta }$, where *R* is the indenter radius and *δ* the indentation depth. Therefore, when considering the scaling analysis, the characteristic time scale for ramp phase of spherical indentation is $\tau_{R}=\frac{Dt_{R}}{R\delta_{M}}$ and indentation with the condition of *Dt_R_* ≪ *Rδ_M_* (i.e. *t_R_* ≪ *L*^2^/*D*) leads to enhanced poroelastic effects. However, achieving infinitely small *t_R_* (*τ_R_* ≪ 1) is not possible experimentally and imposing very high ramp velocities may lead to unwanted oscillations of cantilever/ indenter following the ramp phase and at the initial stages of the hold phase.

To construct a framework to analyze poroelastic curves that considers the effects of finite approach velocities, we first normalized *t_R_* in the curves of Fig. 4(a) and (b) using $\tau_{R}=\frac{Dt_{R}}{R\delta_{M}}$. This normalization did not lead to the collapse of the curves (Fig. 4(c) and (d)) because *F_M_* and *δ_M_* can also vary. However, normalization of *F_M_* using Eq. (1) led to the collapse of all 6 curves in Fig. 4(c) and (d) into a single master curve, Fig. 4(e):

(1) $$F_{M}^{*}=\frac{F_{M}\left(\tau_{R}\right)-F_{M}\left(\tau_{R}=\infty \right)}{F_{M}\left(\tau_{R}=0\right)-F_{M}\left(\tau_{R}=\infty \right)}$$

where $\tau_{R}=\frac{Dt_{R}}{R\delta_{M}}$ is the normalized rise time. When the contact size $\left(a=\sqrt{R\delta }\right)$ is significantly smaller than the hydrogel thickness *d* (semi-infinite condition, i.e. $\frac{\sqrt{R\delta }}{d}≅0$), the Hertz solution gives an accurate estimate of the force–indentation relationship [17,49]:

(2) $$F=\frac{4}{3}\;\sqrt{R}\;\delta^{3/2}\frac{E}{\left(1-\upsilon^{2}\right)}$$

When a load is suddenly applied to a poroelastic material, i.e. $\tau_{R}=0$, since the fluid does not have enough time to redistribute, the volumetric changes are negligible and one can assume near-incompressible conditions, implicating a Poisson ratio of υ=0.5 [45], [46], [47], [48] and $F_{M}\left(\tau_{R}=0\right)=\frac{4}{3}\;\sqrt{R}\;\delta_{M}^{3/2}\frac{E}{0.75}$. Allowing sufficiently large enough rise time, $\tau_{R}=\infty$, results in the full redistribution of the fluid and an equilibrium state. Here, a Poisson ratio of *υ* can be considered for the material's elastic response yielding $F_{M}\left(\tau_{R}=\infty \right)=\frac{4}{3}\;\sqrt{R}\;\delta_{M}^{3/2}\frac{E}{\left(1-\upsilon^{2}\right)}$ . Assuming these two limiting cases and Hertz condition, Eq. (1) takes:

(3) $$F_{M}^{*}\left(\tau_{R}\right)=\frac{\frac{F_{M}\left(\tau_{R}\right)}{\frac{4}{3}\;\sqrt{R}\;\delta_{M}^{3/2}E}-\frac{1}{1-\upsilon^{2}}}{\frac{1}{0.75}-\frac{1}{1-\upsilon^{2}}}$$

On the other hand, the master curve in Fig. 4(e) can be empirically represented via a continuous exponential function Eq. (4):

(4) $$F_{empirical\;}^{*}\left(\tau_{R}\right)=12.5\exp\left(-2.63{\tau_{R}}^{0.12}\right)$$

Therefore, a set of poroelastic parameters can be estimated (*D, E* and υ) considering Eqs. (3) and 4 and solely performing the ramp phase of indentation experiments with different indentation velocities. This framework has been applied to experimentally derive poroelastic parameters. Briefly, initial estimates for a set of (*D, E*, υ) can be made and $F_{M}^{*}\left(\tau_{R}\right)\;$in Eq. (3) can be calculated from experimental data while $F_{empirical\;}^{*}$from Eq. (4) can also be estimated. The set (*D, E*, υ) can be varied until the error *(*$F_{M}^{*}\left(\tau_{R}\right)-F_{empirical\;}^{*})$ is minimized. Our framework implies that poroelastic properties can be estimated by conducting only a few indentation tests with only one data point collected for each approach velocity (i.e. either the maximum force or indentation depth for the prescribed maximum indentation depth or force, respectively). Since the decay of Eq. (4) is exponential, conducting several tests with incremental fold changes in approach velocities would be the most appropriate selection for the set of velocities. Furthermore, depending on the limitations of the instrument and experimental conditions, collecting more data at higher velocities will increase the accuracy for the estimation of poroelastic parameters. However, under our framework it is not essential to collect the data at extremely high approach velocities that are beyond the instrument capabilities.

### 3.4 Experimental validation

We developed a theoretical framework, as a first approximation, using a FEM approach considering indentation of an ideal linear isotropic poroelastic material. In real life experiments, the instrument errors, testing/boundary conditions and other experimental parameters, as well as material conditions and properties (i.e. non-linear and non-poroelastic contributions such as hyperelasticity and intrinsic viscoelasticity), may contribute to the maximum force generated at specified approach velocities. Therefore, to test the applicability of our approach which is based on an approximated linear framework, in the second part of this work we conducted experiments on two different types of hydrogels at both macro and micro scales using two different testing machines. Both microscale AFM and macroscale indentation tests were conducted on agarose and PAAm hydrogels in order to experimentally test our proposed framework which incorporates the effects of approach velocity. While the AFM setup normally allows conducting relaxation tests on very soft materials with a fixed maximum set force (*F_M_*), our macroscale setup enabled us to perform relaxation experiments whilst setting a fixed maximum indentation depth (*δ_M_*).

In microscale experiments, we used small indenters (µm scale) and the AFM setup allowed us to apply and monitor pN and nN forces at extremely high sampling rates (kHz). In macroscale tests, we used large indenters (mm scale) and a uniaxial tester machine that allowed for the application and monitoring of mN range forces at lower sampling rates (10 Hz) which are sufficient to record poroelastic relaxation times at macroscale. The experimental conditions (such as approach velocities, maximum forces and indentation depths) and the range of stiffness for the tested hydrogels, for both AFM experiments and macro-indentation tests, were such that both instruments were sufficiently stiff during the indentation tests. One of the main advantages of our work is to introduce a framework that does not require conducting stress-relaxation tests at extremely fast approach velocities. Our method is based on capturing maximum force or indentation depth at different rise times and not necessarily extremely fast approach velocities. Therefore, one could consider the limitation of their testing machine such as stiffness and sensitivity and tune the experimental parameters (such as size of the indenter, approach velocities, maximum force, indentation depth, etc.) to conduct indentation experiments at such time and length scales that minimize the experimental errors and are relevant to our developed framework.

#### 3.4.1 Micro indentation experiments

**Fig. 5:**
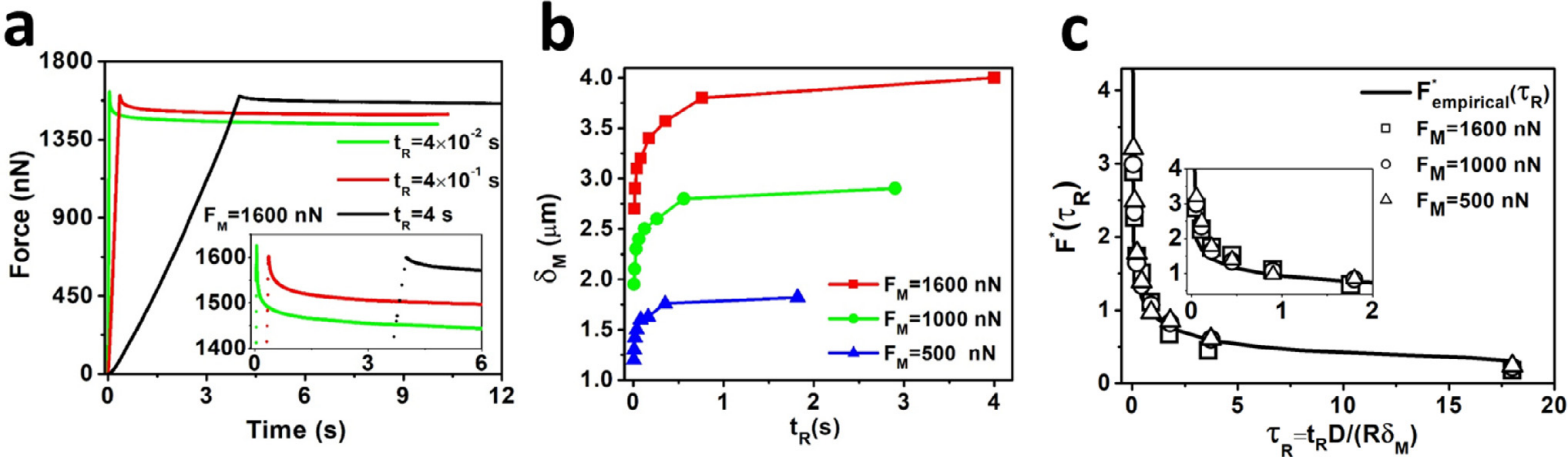
Microscale AFM experiments with a force clamp on 0.6% agarose gel using *R* = 25 µm spherical indentor. (a) Effects of three approach velocities (*V* = 80, 10, 1 µm.*s* ^−^ ^1^) on ramp and hold phases of stress-relaxation experiments. (b) Maximum indentation depth *δ_M_* for *F_M_* = 500, 1000 and 1600 nN is estimated for different approach velocities (*V* = 320, 160, 80, 40, 20, 10, 5, 1 µm s^−1^) and plotted against rise time *t_R_*. (c) The microscale experimental data confirm the theoretical master curve. Poroelastic parameters for all conditions were estimated by comparing Eqs. (3) and 4. Subsequently $F_{M}^{*}\left(\tau_{R}\right)\;$and $F_{empirical\;}^{*}$were estimated using the experimental parameters and plotted against normalized rise time *τ_R_*.

Using low concentration (0.6%) agarose hydrogels and a small probe size of *R* = 25 µm allowed us to conduct microscale AFM indention tests with soft cantilevers in order to extract force–indentation and force–relaxation curves with a good signal to noise ratio. The force–relaxation curves, obtained with a fixed *F_M_* = 1600 nN and three different approach velocities *V* = 80, 10, 1 µm s^−1^ (rise time of *t_R_* = 0.04, 0.4 and 4 s) are plotted in Fig. 5(a). Since the rise time changed from O(10^−2^ s) to O(1 s) significant differences in the shapes of both ramp and hold phases were observed. Next, 24 indentation experiments were conducted by varying *F_M_*=500, 1000, 1600nN and approach velocities *V* = 320, 160, 80, 40, 20, 10, 5, 1 µm s^−1^. For each set of *F_M_* and *V*, the maximum indentation depth *δ_M_* was calculated and plotted against *t_R_* in Fig. 5(b). A set of poroelastic parameters (*D, E, υ*) was obtained by minimizing the differences between Eqs. (3) and (4); yielding *E* = 28 ± 1 kPa, *υ* = 0.12 ± 0.02 and *D* = 4.5 ± 0.8 × 10^−10^m^2^ s^−1^. Considering these sets of poroelastic parameters, Fig. 5(c) shows strong overlap of experimental $F_{M}^{*}\left(\tau_{R}\right)$ derived from Eq. (3) and the presented $F_{empirical\;}^{*}$ in Eq. (4).

Here, poroelastic parameters were obtained by fitting the microscale indentation results on the master curve derived in Section 3.2 with the assumption that the hydrogel is an isotropic linear poroelastic material. The values of elastic modulus are similar to those measured previously for 0.6% agarose hydrogels with high viscosity [50]. However, a significant deviation was found in the diffusion coefficient values compared to those obtained by macro-indentation experiments of [4,50], where the poroelastic diffusion coefficient was estimated to be *D_Agarose-0.6%_* = 6 × 10^−7^m^2^ s^−1^. Part of this inconsistency is due to the inherent differences between macro- and micro-scales experiments since poroelastic responses are length scale dependent. Therefore, in order to measure poroelastic materials with a large fluid permeability (*D* > 10^−9^m^2^ s^−1^), we found that the AFM contact depths and probe diameter must be large enough to capture the true poroelastic effects [22]. To conduct experiments with a significantly larger probe size, a significantly stiffer cantilever is needed to apply large forces and achieve suitable indentation depths. However, we were unable to achieve these conditions for microscale AFM experiments. Therefore, macroscale indentation tests on agarose (with concentrations of 0.6% and 1%) and PAAm hydrogels were next performed.

#### 3.4.2 Macro indentation experiments

**Fig. 6:**
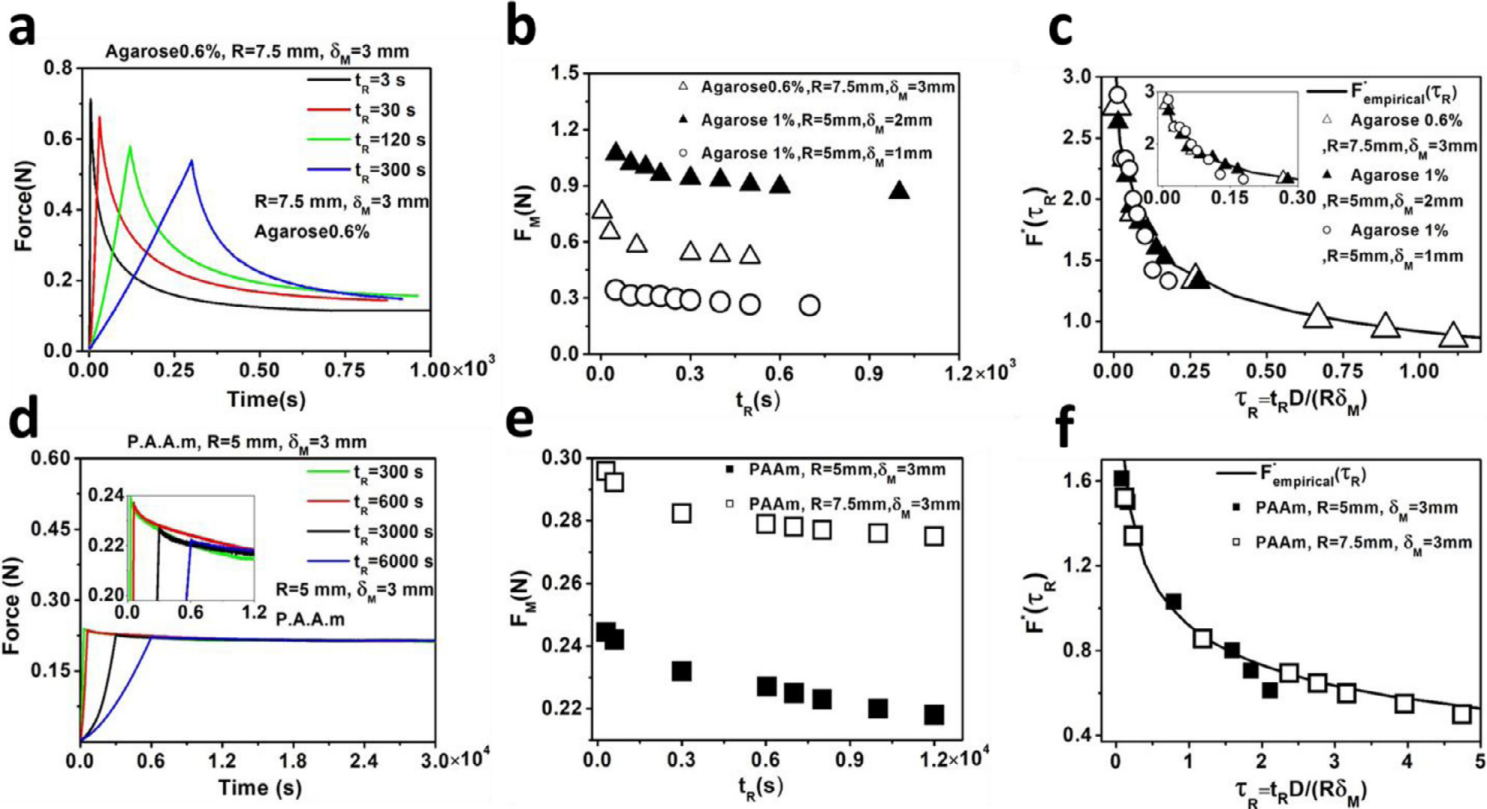
Macroscale indentation experiments with indentation clamp on agarose and PAAm hydrogels using two macro scale spherical indenters (*R* = 5 mm and 7.5 mm). (a, d) Effects of approach velocity in ramp and hold phases of stress-relaxation experiments on 0.6% agarose (*V* = 1, 0.1, 0.025, 0.01) and PAAm (*V* = 0.01, 0.005, 0.001, 0.0005 mm s^−1^). (b, e) Maximum forces *F_M_* on (0.6, 1%) agarose and PAAm for *δ_M_* = 1, 2, 3 mm and *R* = 5, 7.5 mm are estimated for different approach velocities and plotted against rise time *t_R_*. (c, f) The Macroscale experimental data confirm the theoretical master curve. Poroelastic parameters for three gels at different conditions were estimated by comparing Eqs. (3) and (4). Subsequently $F_{M}^{*}\left(\tau_{R}\right)\;$and $F_{empirical\;}^{*}$were estimated using the experimental parameters and plotted against normalized rise time *τ_R_*.

Because it is easier to control large forces and displacements in our indenter machine, we could achieve a precise target indentation depth in our macro scale tests, as opposed to AFM experiments, where a target displacement cannot be achieved due to the arbitrary nature of the AFM's displacement coordinate system and the absence of a closed loop control system during cantilever deformation. Macro indentation tests were performed on PAAm and agarose (0.6, 1%) hydrogels with *t_R_* in the range of a few seconds to a couple of hours, which resulted in relaxation times in the range of a few mins to ∼10 h. Force–relaxation curves were conducted on agarose hydrogels (0.6% and 1%) by setting maximum indentation depths of *δ_M_*=1, 2, 3 mm, three sets of velocities *V* = 1, 0.1, 0.025, 0.01 (Fig. 6(a)), *V* = 0.04, 0.014, 0.01, 0.005 (Fig. S6a) and *V* = 0.02, 0.007, 0.005, 0.004 mm.*s* ^−^ ^1^ (Fig. S6b) and two indenter sizes (*R* = 5 mm and 7.5 mm). The considered indentation depths and approach velocities led to rise times in the range of 3 s to 400 s and maximum forces from 0.296 N to 1.06 N. For agarose indentations with large indenters (mm scale), relaxation occurs over a timescale of ∼hours while in microscale indentations (AFM tests with *R* = 25 µm) relaxation times were only in the order of few seconds (Fig. 5(a)).

Experiments on PAAm hydrogels were conducted with a maximum indentation of *δ_M_* = 3 mm, approach velocities of 0.01, 0.005, 0.001, 0.0005 mm s^−1^ and two indenters sizes of *R* = 5 (Fig. 6(d)) and *R* = 7.5 mm (Fig. S6c). Increasing the PAAm hydrogel concentration, crosslinking density and structure resulted in a less permeable material [50,51] and, therefore, significantly greater approach velocities were required to pressurize the interstitial fluid and capture relaxations due to poroelastic effects. Longer relaxation times for PAAm (in the order of ∼10 h) were observed compared to agarose (in order of ∼30 min). Furthermore, the force under the largest velocity condition relaxes to its maximum at 85% for 0.6% agarose, 50% for 1% agarose and ∼15% for PAAm, indicating significant mechanical differences between agarose at different concentrations and PAAm hydrogels.

To estimate the poroelastic parameters using our new framework, the estimated maximum force *F_M_* was calculated and plotted against the rise time for agarose in Fig. 6(b) and PAAm in Fig. 6(e). Subsequently, by minimizing the root mean square error, the poroelastic parameters were extracted and experimental data was fitted to our empirical master curve, as shown in Fig. 6(c) for agarose and Fig. 6(f) for PAAm. For 0.6% agarose, we found *E_agarose-0.6%_* = 21.25 ± 4.1 kPa, *υ_agarose-0.6%_* = 0.24 ± 0.07 and *D_agarose-0.6%_* = 5 ± 0.78 × 10^−8^m^2^ s^−1^, whilst for 1% agarose *E_Agarose-1%_* = 66.56 ± 10.4 kPa, *υ_Agarose-1%_* = 0.22 ± 0.03 and *D_Agarose-1%_* = 2.03 ± 1.03 × 10^−9^ m^2^ s^−1^. Moreover, for PAAm the estimated poroelastic parameters are *E_PAAm_* = 11.24 ± 1.6 kPa, *υ_PAAm_* = 0.425 ± 0.05 and *D_PAAm_* = 6.43 ± 1.18 × 10^−9^ m^2^ s^−1^. We found that 1% agarose is significantly stiffer than 0.6% agarose and PAAm, and also that it has significantly lower effective diffusivity due to an increase in hydrogel concentration and crosslinking density in the material [52,53]. Furthermore, for 0.6% agarose [50], our estimated elastic moduli from micro and macro indentation tests are similar while the calculated poroelastic diffusion constants are significantly different. Indeed, the estimated values from our macro indentation tests are consistent with values reported in the literature [4,51]. These findings indicate that length scale is a critical factor when determining the poroelastic properties.

## 4 Further discussion

Here, we proposed and tested a novel framework to extract poroelastic properties via indentation tests. However, there are some limitations associated with both FEM and experimental aspects of our work. In the FEM, the contact between the indenter and the hydrogel was considered to be frictionless and impermeable. In addition, like many other recent works [21], [22], [23], [24],^34^,^35^,[40], [41], [42],54], we considered the solid phase to behave like an ideal linear isotropic elastic material under small strain conditions. While these considerations are quite simplistic for many soft and biological materials, our framework could minimally address the effects of ramp speed and determine the poroelastic parameters solely based on ramp phase of force–indentation experiments. Furthermore, application of our framework to experiments requires optimized instrument settings to minimize the inertia effects, sufficiently stiff systems and a load sensor with good resolution to record the maximum overshoot.

While investigating effects of large deformation and material nonlinearity is beyond the scope of our work, recent research [40], [41], [42] suggest that for *δ* ≪ *R* and √*δ_R_*/*h* < ∼0.1 (where h is sample thickness) conditions, the linear isotropic conditions are valid and the effects of substrate thickness can be negligible [18, 22]. Furthermore, for up to *δ/R* ∼ 0.6 the Hertz load displacement relationship still holds and, therefore, our methodology results in a good estimation of poroelastic parameters as a first approximation. To compare our results with a hyperelastic neo-Hookean model, we conducted further FE simulations and found that the force–indentation and force–relaxation curves are very similar for both linear elastic and hyperelastic cases with less than 1% differences in the maximum forces (Figs. S8a, b). Furthermore, we avoid the application of large strains so as to minimize the hyperelastic and plastic effects. For macroindentation of agarose gels, we visualized the surface of the gel after removing the indenter and noticed that, following retraction of the indenter, the indented area returned approximately to its original shape over a few minutes. This confirmed the dominance of the elastic regime in our experiments.

Our simulations are sensitive to the number of mesh elements and boundary conditions. To test the level of mesh sensitivity, we ran further mesh sensitivity analyses for a few simulations and found that the normalized curves were weakly dependent on the number of mesh elements (Fig. S10). Furthermore, for the Poisson ratios of *υ* *=* 0.2 to *υ* *=* 0.45 which is the expected range for most biomaterials, we found that the normalized force–relaxation curves (Fig. S9d) as well as the master curve (Fig. S9e) exhibit a significantly weak dependency to the Poisson ratio which is consistent with previous reports [47,54].

We found that the Poisson ratio of the agarose gel was significantly smaller than PAAm. The physical role of Poisson ratio during indentation of poroelastic materials implies that when *υ* → 0.5, there is no net movement of the interstitial fluid out of the hydrogel following indention, i.e. equilibrium is reached, whereas, when *υ* << 0.5 some interstitial fluid has to squeeze out of the hydrogel [47]. According to the theory of linear isotropic poroelasticity [23,^27^,28], the basic independent poroelastic parameters are elastic modulus of drained solid phase *E*, Poisson ratio and hydraulic permeability *K* which are correlated through poroelastic diffusion constant via *D*/(*K E*) = (1– *υ*)/((1–2 *υ*).(1+ *υ*)). Considering this relationship, the *D*/(*K E*) ratio is highly sensitive to the Poisson ratio for *υ* *>* *0.4*. Therefore, from a macroscopic point of view, the Poisson ratio is highly coupled to the elastic modulus and poroelastic diffusion coefficient and it is difficult to estimate the exact values of Poisson ratio (up to second digits for *υ* *>* *0.4*) from indentation experiments [11,^22^,[55], [56], [57]. However, our experimentally approximated Poisson ratios for both gel systems (*υ_agarose-0.__6_*_%_ = 0.24 ± 0.07 and *υ_PAAm_* = 0.425 ± 0.05) are very close to the ones reported in the literature [11,^22^,[58], [59], [60] and we found that *υ_agarose-0.6%_* <  *υ_PAAm_* consistent with other reported data. These data suggest that, from the macroscopic point of view, it would be easier to squeeze the fluid out of the agarose gel. From a microscopic point of view, the physical interpretation of Poisson ratio is mainly related to the shape of the polymeric unit, its structure, the type and density of crosslinking, as well as the fraction of free versus bound water within the gel pore. Therefore, consistent with this physical nature, the different Poisson ratio for the two gel systems originates from the differences in the microstructural organization of the two gels.

To compare our estimated diffusion coefficient with previously developed methods that solely consider stress-relaxation curves under ideal ramp conditions (i.e. fast approach velocity), we normalized two force relaxation curves from our experiments on PAAm gels (Fig. 6(d)) using the methodology introduced by Hu et al. [17]. The normalization of force–relaxation curves with two different approach velocities did not lead to the collapse of relaxation curves into a single curve (Fig. S7) and also the proposed master curve did not provide a good fit for the normalized curves, obviously because the method of Hu et al. does not consider the effect of approach velocity. Furthermore, we estimated diffusion coefficient using their master curve and found that while at small rise times the estimated diffusion coefficient is close to our estimated values (*D_PAAm_* = 4.12 ± 1.2 × 10^−9^ m^2^ s^−1^ for *t_R_* = 300 s compared to our estimation of *D_PAAm_* = 6.43 ± 1.18 × 10^−9^ m^2^ s^−1^), at larger rise times the diffusion coefficient is an order of magnitude smaller (*D_PAAm_* = 8.32 ± 0.4 × 10^−10^ m^2^ s^−1^ for *t_R_* = 600 s). Taken together, these results indicate that while previous methodologies are significantly sensitive to the rise time, our framework provides better estimation of poroelastic parameters in general.

One further caveat of our work is that we assumed that time-dependent behavior originates from a single physical mechanism, i.e. poroelastic effects. For the agarose hydrogel there were some discrepancies, particularly for microscale experiments, where the inherent viscoelastic timescale may fall within a range close to poroelastic timescales. Dissecting the contribution of different relaxation times originating from different relaxation mechanisms, including inherent viscoelasticity, plasticity and poroelasticity, is a major challenge for the characterization of soft hydrated materials. However, since a major hallmark of poroelasticityis the lengthscaledependency of the timescale, in this work and particularly in macroindentation tests we selected the lengthscale (contact area of the indenter) such that poroelastic mechanisms dominate the ramp response (i.e. pressurization) and relaxation timescale.

## 5 Summary and conclusions

**Fig. 7:**
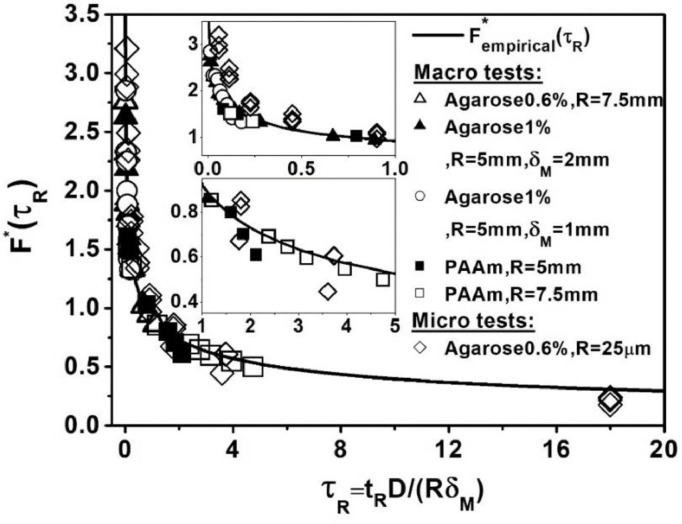
All micro and macro experiments on agarose (0.6%, 1%) and PAAm hydrogels match the master curve of our suggested framework obtained by FEM. Poroelastic parameters for these gels at different conditions were estimated by comparing Eqs. (3) and 4. Subsequently $F_{M}^{*}\left(\tau_{R}\right)\;$and $F_{empirical\;}^{*}$were estimated using the experimental parameters and plotted against normalized rise time.

We present here a new generalized indentation framework to extract the poroelastic properties of materials from indentation tests using spherical indenters at different indentation approach velocities. Fig. 7 summaries the fit of all experimental micro (Fig. 5) and macro (Fig. 6) tests to the master curve that we obtained from FEM.

The collapse of all normalized simulated and experimental curves (normalized maximum force versus normalized rise time) into a single master curve (Figs. 4(e) and 7) indicates a unique behavior of poroelastic materials. Using FEM, we extracted a master curve (Eq. (4)) for the ramp phase of tests which makes our methodology readily usable and quick to analyze without the need for computational simulations. Normally, to analyze relaxation curves a precise feed-back control loop is essential, especially at very high approach velocities, to avoid unwanted oscillations. Our methodology allows for indentation tests to be carried out by AFM or other instruments on material surfaces without the need to monitor and control the relaxation phase. Fig. 7 summaries the fit of all experimental micro (Fig. 5) and macro (Fig. 6) tests to the master curve that we obtained from FEM. We found that poroelastic properties can be determined through micro/macro indentation tests by applying the master curve method derived solely from force–indentation curves. For PAAm, these findings are in strong agreement with those of [22,61]. The values of elastic modulus at 1% agarose obtained here are similar to those obtained by Normand [50] and a good agreement exists between the diffusion constants obtained with 1% agarose and the results obtained by the authors in Refs. [4,51]. Taken together, these findings confirm that our novel framework is appropriate for determining the poroelastic characteristics of hydrogels via micro and macro indentation experiments.

## Declaration of Competing Interest

The authors declare that they have no known competing financial interests or personal relationships that could have appeared to influence the work reported in this paper.

## Appendix Supplementary materials

Image, application 1
