# Supplementary material for: A new framework for characterization of poroelastic materials using indentation
Source: Acta Biomater. 2020 Jan 15;102:138–48. doi: 10.1016/j.actbio.2019.11.010 (PMC6958526; doi:10.1016/j.actbio.2019.11.010)
Supplement: Supplementary file 1 [file mmc1.docx]

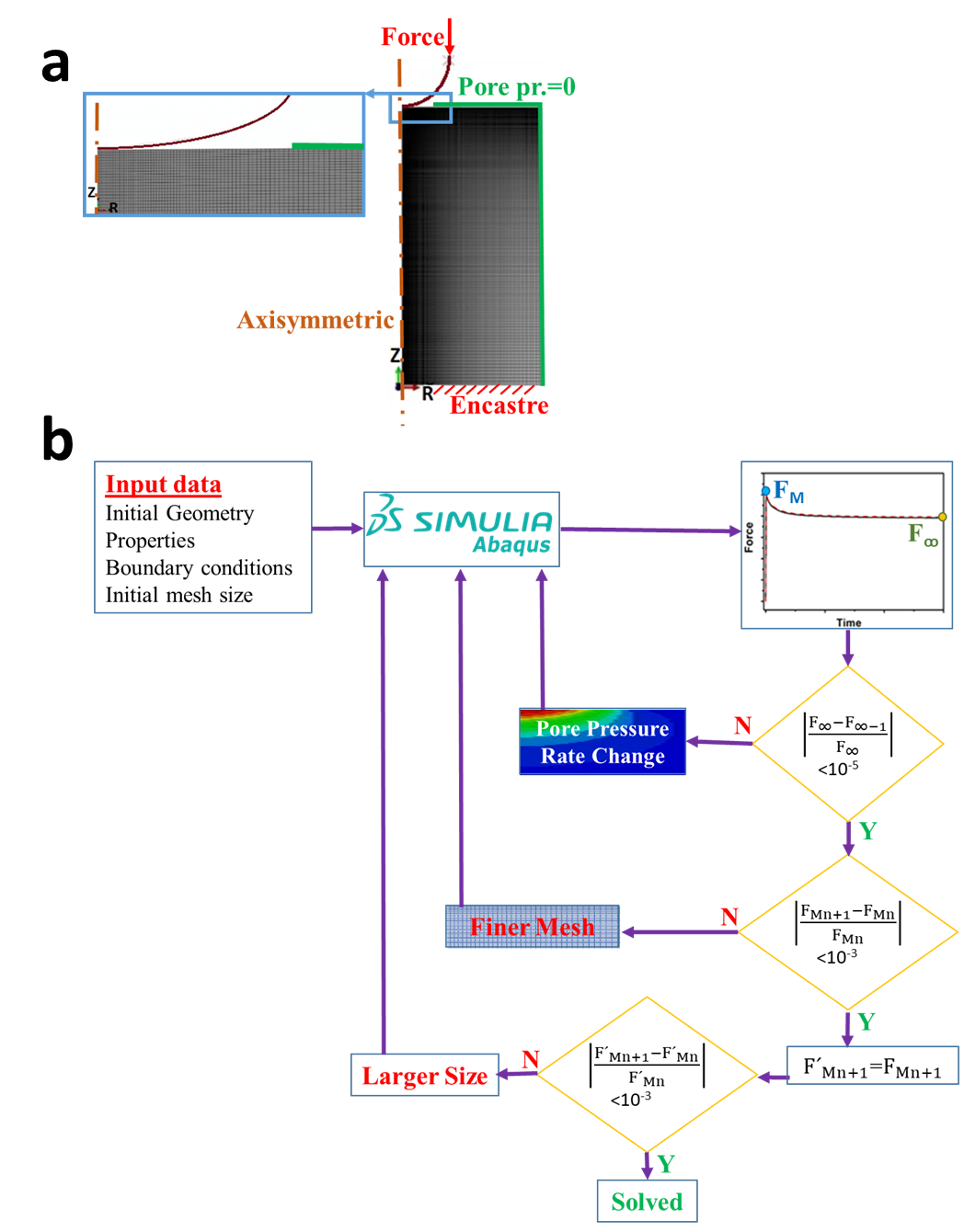


**Fig. S1** (a) Geometry and boundary conditions of the discretized model. The pore pressure was set to zero for both the top and side surfaces of the specimen (excluding the indenter contact surface) to simulate free draining of the interstitial fluid from the porous network. (b) FEM methodology flowchart. For every *t_R_*, in the indentation depth *δ_M_* and maximum force control cases, the maximum indentation force (*F_M_(t_R_)*) and indentation depth (*δ_M_(t_R_)*) functions are calculated, respectively. Convergence of the FEM program for each case is guaranteed by comparing the pore pressure rate, the relaxation force (*F_ꚙ_*) error difference and the maximum indentation force (*F_M_*) error difference in their independent loops.

**Table S1**. Poroelastic properties of hydrogel sample applied in FEM [22]

| Indentation depth,  $\delta\left( \mu m \right)$ | Shear modulus,  $G \left( \mathrm{kPa} \right)$ | Diffusivity,  $D \left( {m^{2}}/s \right)$ | Poisson ratio,  $\upsilon$ | Fluid viscosity,  $\eta\left( N.s/m^{2} \right)$ |
| --- | --- | --- | --- | --- |
| 100 $\left( R=10mm \right)$ | 6.6 | $2.03\times{10}^{-10}$ | 0.41 | $1.0\times{10}^{-3}$ |
| 8 $\left( R=22.5\mu m \right)$ | 6.21 | $1.70\times{10}^{-10}$ | 0.39 | $1.0\times{10}^{-3}$ |

**Table S2**. Poroelastic properties of neo-Hookean model in FEM [22,43]

| Indentation depth,  $\delta\left( \mu m \right)$ | Material constant,  $C_{10} \left( \mathrm{kPa} \right)$ [43] | Material constant,  $D_{1} \left( \mathrm{kPa} \right)^{-1}$ [43] | Diffusivity,  $D \left( {m^{2}}/s \right)$ [22] | Fluid viscosity,  $\eta\left( N.s/m^{2} \right)$ [22] |
| --- | --- | --- | --- | --- |
| 100 $\left( R=10mm \right)$ | $3.25$ | $5.89$ | $2.03\times{10}^{-10}$ | $1.0\times{10}^{-3}$ |

**
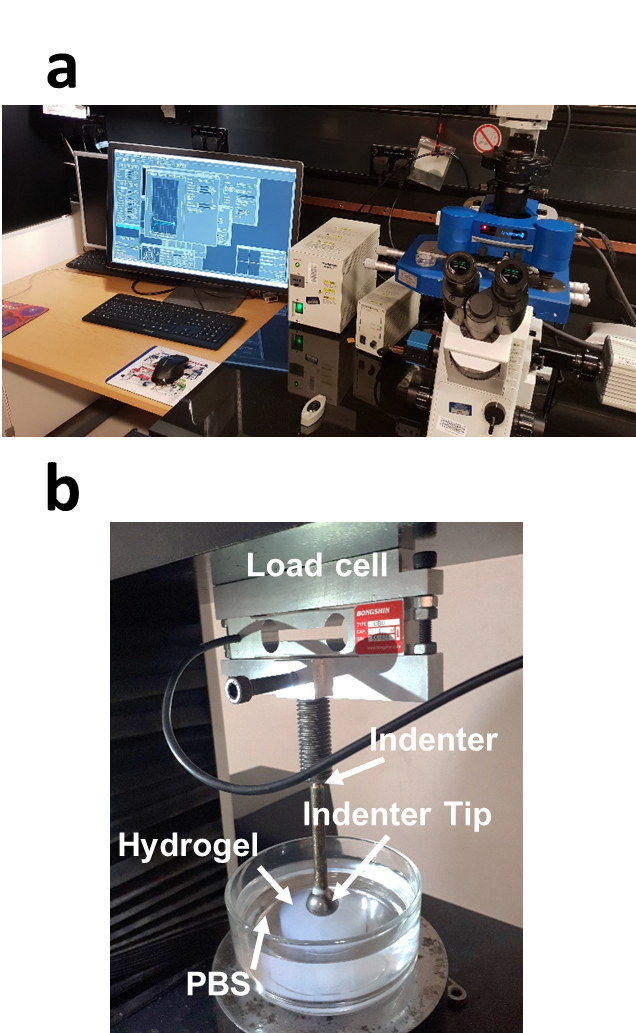
**

**Fig. S2** The microscale indentation experiments were performed by AFM and the macroscale indentation experiments were carried out by applying a uniaxial tensile tester machine. (a) In the microscale indentation setup, 25μm radius glass beads were used to indent the softest hydrogels (0.6% agarose) submerged in PBS solution. (b) In the macroscale indentation setup, a rigid stainless steel spherical indenter radius of either *R*=5, 7.5 or 10mm was applied on the 6% PAAm, 0.6% and 1% agarose hydrogels, which were fully submerged in PBS during all indentation experiments.


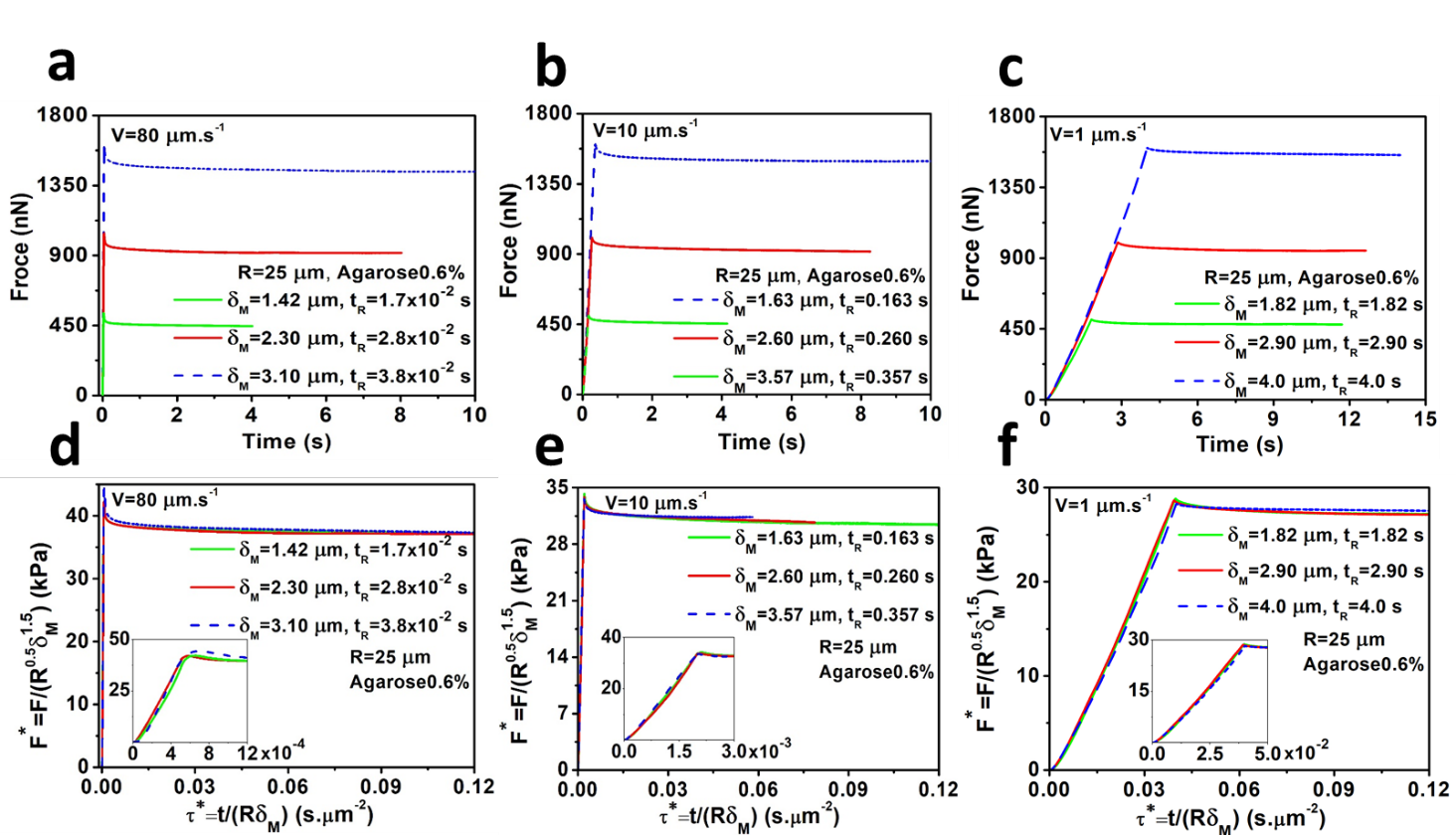


**Fig. S3** The microscale force-indentation and -relaxation curves and their normalization (0.6% agarose and indenter size *R*=25µm). Force-indentation and -relaxation under constant approach velocity for different indentation depths and three different velocities (a) *V*=80µm.s^-1^, (b) *V*=10µm.s^-1^, and (c) *V*=1µm.s^-1^, and three target forces (d) 500nN, (e) 1000nN, and (f) 1600nN. The indentation depth $\delta_{M}$ and rise time $t_{R}$ were varied proportionally to keep the approach velocity $V={\delta_{M}}/{t_{R}}$ constant leading to the collapse of all curves into a single curve after implementing appropriate force and time normalizations.


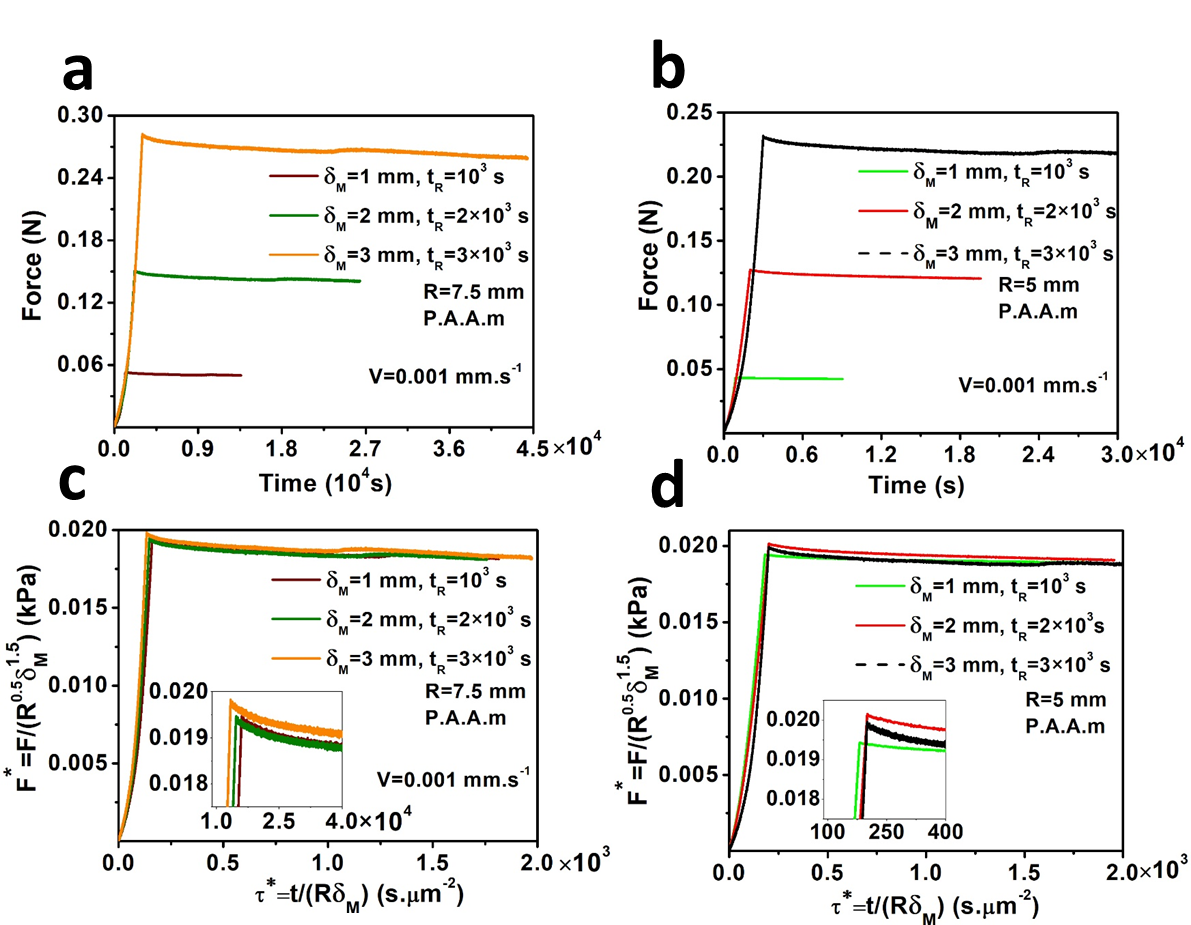


**Fig. S4** The macroscale force-indentation and -relaxation curves and their normalization (PAAm). (a,b) Force-indentation and -relaxation under constant approach velocity *V*=0.001mm.s^-1^ for different indenter sizes, *R*=5, 7.5mm and *δ_M_*=1, 2, 3mm; (c,d) The indentation depth $\delta_{M}$ and rise time $t_{R}$ were varied proportionally to keep the approach velocity $V={\delta_{M}}/{t_{R}}$ constant leading to the collapse of all curves into a single curve after implementing appropriate force and time normalizations.


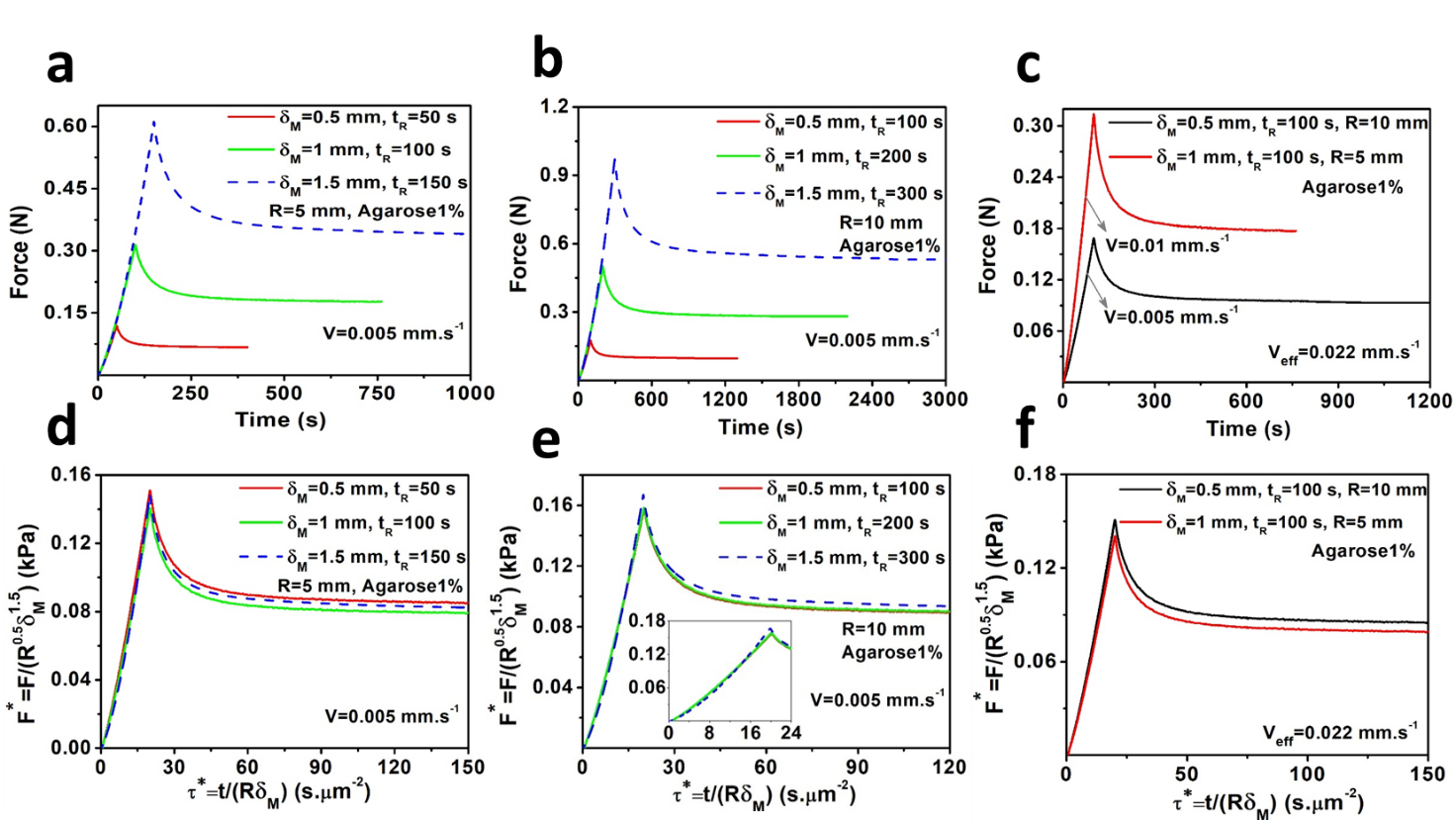


**Fig. S5** The macroscale force-indentation and -relaxation curves and their normalization (1% agarose). (a,b) Force-indentation and -relaxation under constant approach velocity for different indenter sizes, *R*=5, 10mm and two velocities, *V*=0.01, 0.005mm.s^-1^; (c) Effects of varying indentation depth$\delta_{M}=0.5, 1\mathrm{mm}$, velocity *V*=0.01, 0.005mm.s^-1^ and indenter radius *R*=10, 5mm. (d,e) The indentation depth $\delta_{M}$ and rise time $t_{R}$ were varied proportionally to keep the approach velocity $V={\delta_{M}}/{t_{R}}$ constant leading to the collapse of all curves into a single curve after implementing appropriate force and time normalizations. (f) Only curves with the same ${V_{eff}=\surd(R\delta_{M})}/{t_{R}=0.022}mm.s$^-1^ were collapsed on each other after normalization.


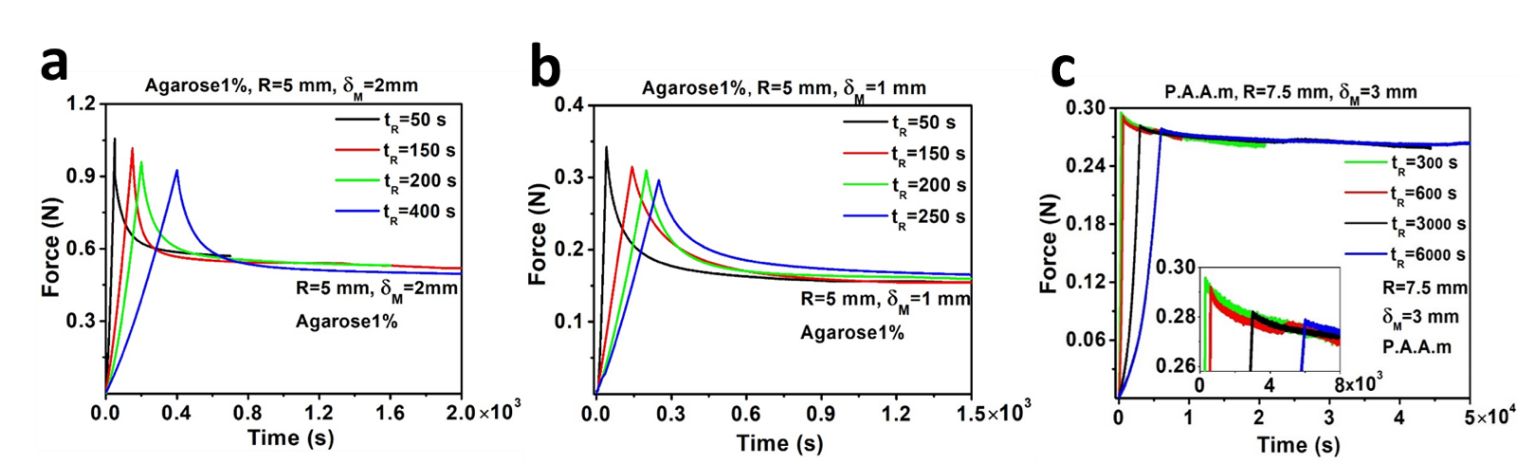


**Fig. S6** Macroscale indentation experiments with indentation clamp on 1% agarose and PAAm hydrogels. (a,b) Effects of approach velocity in ramp and hold phases of stress-relaxation experiments on 1% agarose with two different set maximum indentation depths *δ_M_* =2, 1mm and same *R*=5mm. (c) Effects of approach velocity in ramp and hold phases of stress-relaxation experiments on PAAm with *δ_M_* =3mm and *R*=7.5mm.


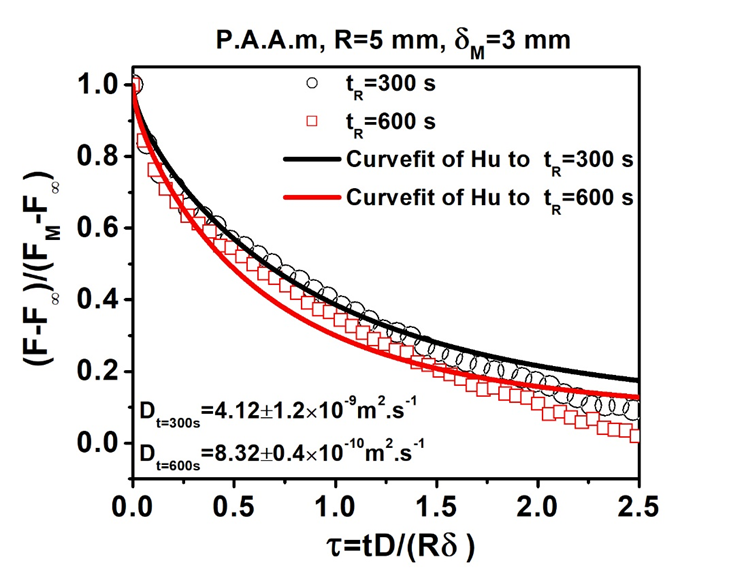


**Fig. S7** Effect of approach velocity on normalization of the force relaxation curves (for PAAm hydrogels with indenter size *R*=5mm and indentation depth *δ_M_*=3mm, Fig.6d). The relaxation curves were considered to start at t=0 and normalized using [*F(t)*-*F_ꚙ_*]/[(*F_M_*- *F_ꚙ_*]. The diffusion constant was found by finding the best fit to the master curve introduced in [17] and the time was also normalized using *tD*/*Rδ*.


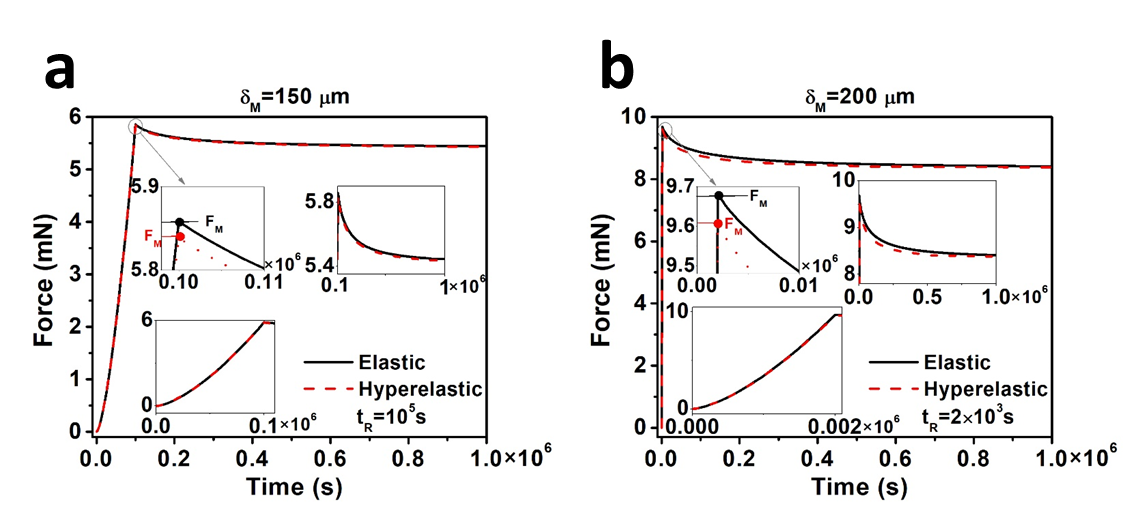


**Fig. S8** Effects of non-linear elasticity: The force-indentation and force-relaxation curves for indentation depths of (a) *δ_M_*=150μm and rise time *t_R_*=2×10^3^s and (b) *δ_M_*=200μm and rise time 10^5^s (with indenter of size *R*=10mm) for isotropic linear and neo-Hookean hyperelastic models.


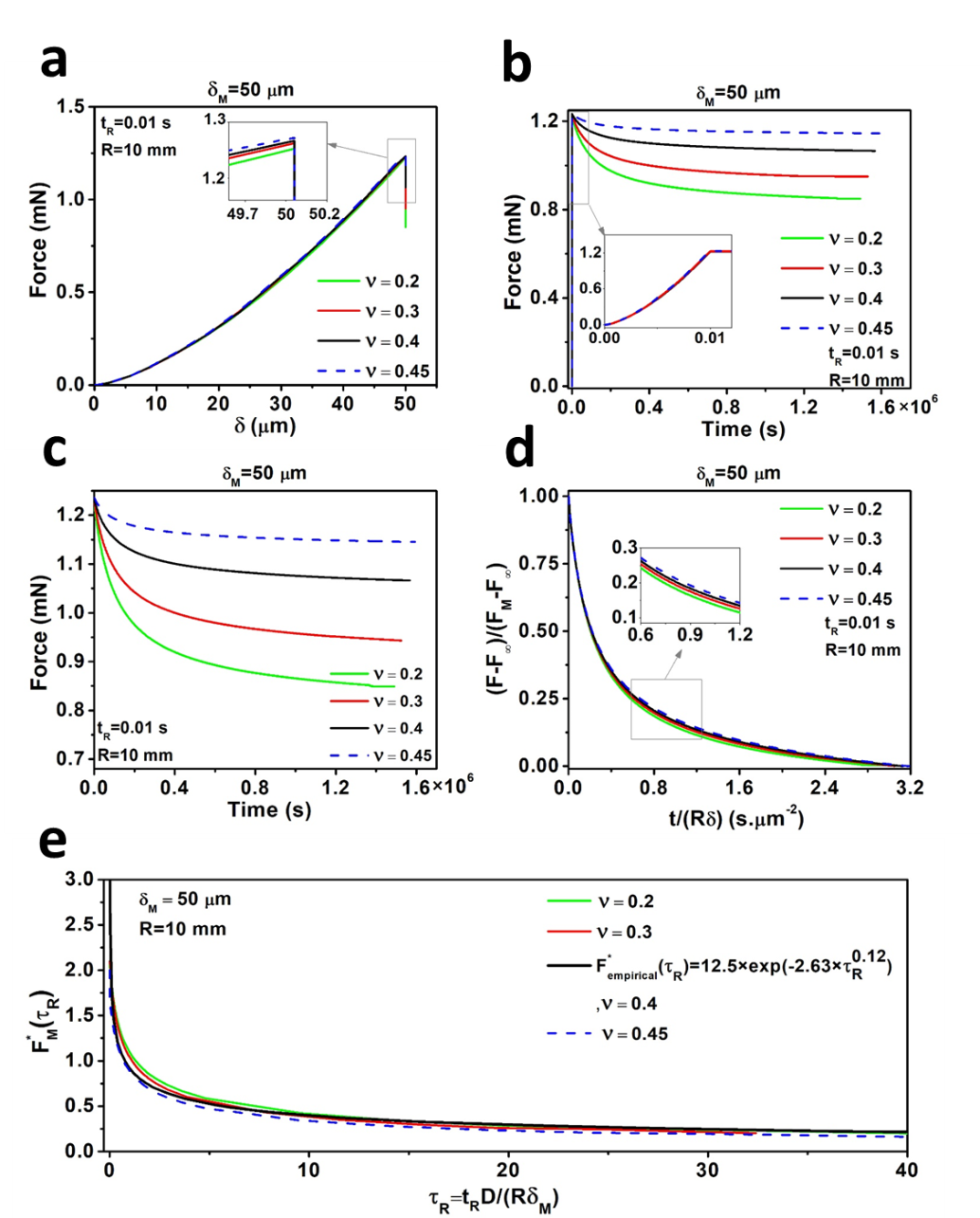


**Fig. S9** Effects of Poisson ratio on force-indentation (a) and force-relaxation (b) curves generated from FEM considering four Poisson ratios of *υ*=0.2, 0.3, 0.4 and 0.45. (c) Only the force-relaxation part of the curves in (b) were considered with the initial time of relaxation curves set to zero. (d) Normalization of the force-relaxation curves in (c) with the methodology introduced in [17,18,22]. In a-b, maximum indentation depth is *δ_M_*=50µm and rise time *t_R_*=0.01s. (e) Effect of the Poisson ratio on our proposed master curve (equation 4).


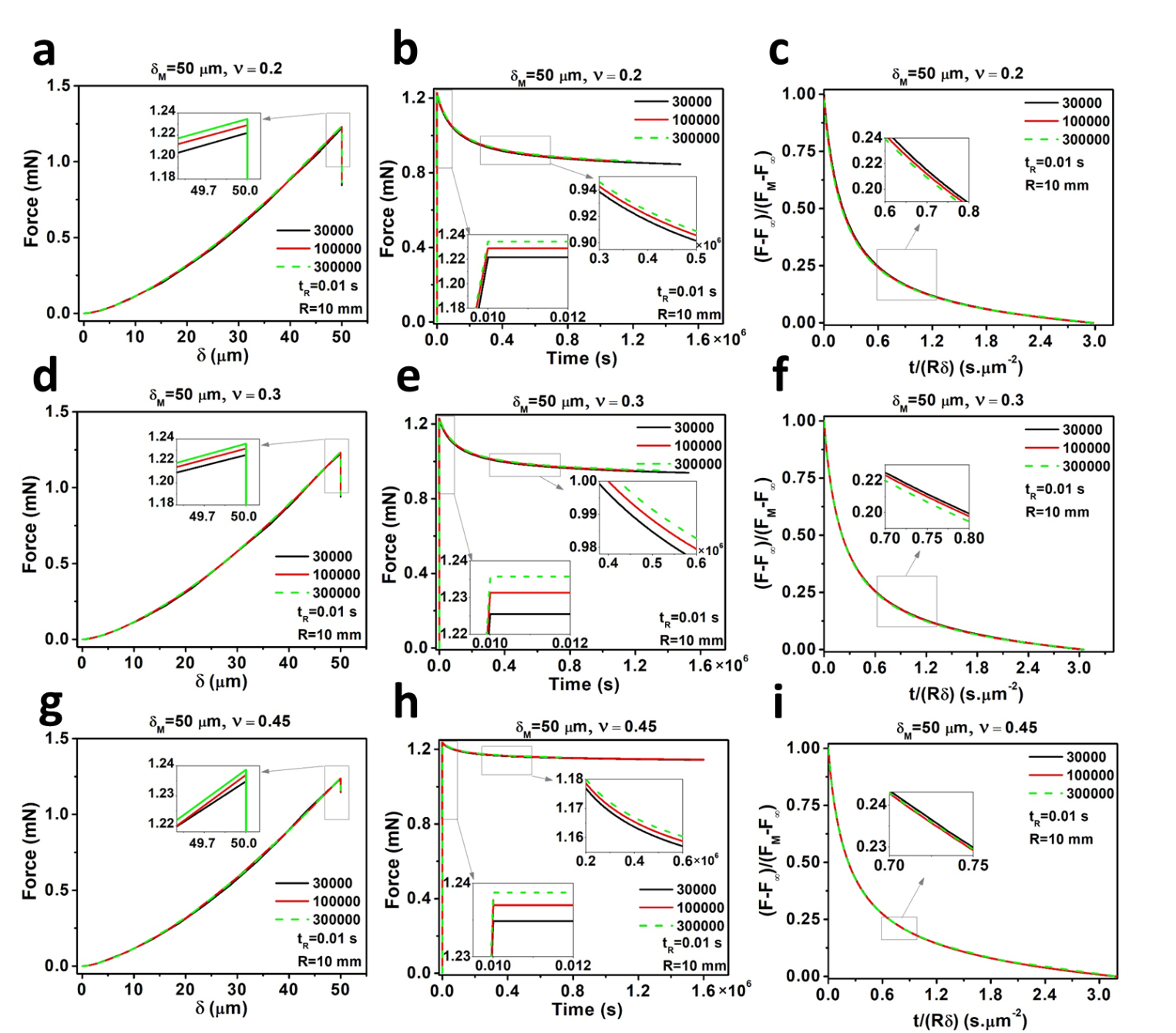


**Fig. S10** Effect of number of mesh elements on force-indentation (a,d,g) and force-relaxation (b,e,h) curves generated from FEM. Three number of mesh elements 30000, 100000, 300000 for three Poisson ratio *υ*=0.2, 0.3 and 0.45 were considered. (c,f,i) Normalization of the force-relaxation curves (hold phase) with the methodology introduced in [17,18,22]. For all three values of *υ*, the shape of both F-δ, F-t and the normalized curves are weakly dependent on the number of mesh elements.
